# Associations of Serum Trace-Element Concentrations with Selected Blood Biomarkers: A Secondary Cross-Sectional Analysis of the SPES Cohort

**DOI:** 10.3390/toxics14090824

**Published:** 2026-09-17

**Authors:** Carlo Buonerba, Paolo Montuori, Ernesto Esposito, Maria Triassi, Pellegrino Cerino

**Affiliations:** 1 Department of Public Health, University of Naples Federico II, 80131 Naples, Italy; 2 Department of Health and Healthcare Services, Directorate-General of the Calabria Region, 88100 Catanzaro, Italy

**Keywords:** human biomonitoring, serum trace-element concentrations, environmental epidemiology, LDL cholesterol, neutrophil-to-lymphocyte ratio, systemic immune–inflammation index, cross-sectional study

## Abstract

Trace elements have nutritional and toxicological roles relevant to lipid metabolism and inflammation. In a secondary cross-sectional study, we examined 19 serum elements and blood biomarkers in 4026 adults from the SPES community biomonitoring programme in Campania, Italy. Low-density lipoprotein cholesterol (LDL-C) and neutrophil-to-lymphocyte ratio (NLR) were co-primary outcomes; systemic immune–inflammation index (SII) was secondary. Adjusted models used multiple imputation and false-discovery-rate (FDR) correction. Selenium, copper and zinc showed positive LDL-C associations among directly quantified concentrations, whereas the mercury association depended on assay availability and quantification. Models with untransformed LDL-C estimated differences of approximately 1–4 mg/dL per element-specific interquartile increase in log concentration. Copper and zinc estimates were smaller in an exploratory joint model. For mercury, LDL-C was higher with quantifiable rather than below-quantification concentrations, but the gradient within the quantified range was uncertain. All four primary-model LDL-C associations met both outcome-specific and combined co-primary FDR thresholds; no primary linear NLR association did. Copper, cobalt, molybdenum and antimony were associated with higher SII after outcome-specific FDR correction, with less consistent results across sensitivity analyses. These modest LDL-C differences warrant prospective investigation; the cross-sectional associations do not establish causal or individual clinical effects.

## 1. Introduction

Lipid metabolism and systemic inflammation contribute to the development of cardiovascular disease, while inflammatory processes also participate in carcinogenesis [1,2]. Blood biomarkers related to these processes have therefore attracted interest in population research, including studies of individuals without previously diagnosed disease. Higher neutrophil-to-lymphocyte ratio (NLR) and systemic immune–inflammation index (SII), two nonspecific indices derived from routine blood counts, have been prospectively associated with incident cancer and cardiovascular disease in population cohorts [3,4,5,6,7]. Low-density lipoprotein cholesterol (LDL-C) has an established causal role in atherosclerotic cardiovascular disease and is a principal target of prevention [8,9,10]. These findings provide a rationale for investigating the nutritional and environmental factors associated with variation in lipid and blood count biomarkers in the general population.

Trace elements intersect with these biological domains through their physiological functions and toxicological effects. Essential elements, including selenium, copper, zinc and iron, participate in enzymatic activity, redox regulation and cellular metabolism; their circulating concentrations reflect nutritional status and physiological regulation [11,12,13,14,15]. Exposure to nonessential contaminants such as lead, cadmium and arsenic has been associated with cardiovascular outcomes, with oxidative stress, endothelial dysfunction and inflammation implicated in their effects [16,17,18,19,20]. Human biomonitoring complements environmental measurements by assessing element concentrations in biological specimens [21]. Studying these concentrations alongside lipid and blood count measurements can therefore inform both nutritional and environmental epidemiology.

Human studies have reported associations between serum selenium and lipid concentrations and between serum copper and lipid profiles [22,23]. Associations between blood metal concentrations and SII have also been described [24]. These findings raise questions about how association patterns extend across elements and biological domains in populations with different nutritional and environmental characteristics. Examining a broad serum panel against both lipid and blood count outcomes within the same community cohort allows these relationships to be characterized under a common sampling and analytical framework.

The Exposure Study on Susceptible People (SPES) is a community biomonitoring programme conducted across areas of differing environmental pressure in Campania, southern Italy. The programme was developed in a regional setting characterized by industrial and urban pressures and longstanding concerns about waste disposal and burning in some areas [25]. A previous analysis of the programme documented geographical variation in serum element concentrations [25]. The availability of concurrent element measurements, routine laboratory data and information on individual characteristics provides an opportunity to extend that geographical characterization to the relationships between circulating elements and biochemical and haematological variation among community residents.

We therefore conducted a secondary cross-sectional analysis of associations between 19 serum elements and selected blood biomarkers in 4026 SPES participants. LDL-C and NLR were the co-primary outcomes, with SII as the key secondary outcome; platelet-to-lymphocyte ratio (PLR), red cell distribution width (RDW), glucose and homocysteine were secondary or exploratory outcomes. The outcome hierarchy was specified before modelling. We used a discovery-oriented approach to characterize the direction and magnitude of element-specific associations, without prespecified directional hypotheses for individual elements.

## 2. Materials and Methods

### 2.1. Study Design and Participants

This secondary cross-sectional analysis used data from SPES, a community biomonitoring programme conducted in Campania in 2016–2017. Municipalities formed 21 environmental pressure clusters, with recruitment targets allocated 4:2:1 across high-, medium- and low-pressure areas [25]. Potential participants were randomly selected from municipal registries and contacted by mail, call-centre follow-up and territorial recruitment units [25,26,27]. Protocol eligibility specified ages 20–49 years, both sexes, informed consent, no reported history of HCV, HBV or HIV infection and at least 10 continuous years of municipal residence [25]. Excluding 17 of 4205 enrolees recruited outside the designated area left 4188 records from 174 municipalities. The data required to calculate an invitation-based participation rate and systematically compare participants with nonparticipants were not collected; registry recruitment alone does not establish representativeness. Reporting follows STROBE and STROBE-ME [28,29]; participant flow (Figure S8) and the combined checklist accompany the Supplementary Materials.

The primary cohort additionally required known negative responses to four non-imputed self-reported physician-diagnosed disease-history items in the self-administered questionnaire: tumour, myocardial infarction, angina and stroke. The tumour item did not establish validated malignant-cancer status. Of 4188 participants, 109 (2.6%) reported at least one history and 53 (1.3%) had incomplete information (51 nonresponders; 2 partial responders), leaving 4026: 96.1% of the parent dataset and 97.4% of 4135 with all histories known. Restriction aimed to reduce distortion by diagnosed disease, treatment or behavioural change but may introduce selection or survivor bias. Sensitivities relaxed or strengthened these restrictions, including all participants with known histories irrespective of positive history. Primary-cohort characteristics are summarized in Table 1.

A structured physical examination was performed before biospecimen collection. For this secondary analysis, the examination and self-reported questionnaire information provided clinical context but could not systematically exclude every acute infection, inflammatory or haematological disorder, recent operation or treatment effect capable of influencing blood counts.

The analysis sample size was fixed by the available parent cohort; no separate prospective sample-size calculation was performed.

### 2.2. Serum Trace-Element Measurements

Participants were instructed to fast for at least eight hours, and study personnel checked fasting status before venepuncture, as described in the published SPES field procedure [27]. The questionnaire, clinical assessment and blood collection took place at the same enrolment encounter. Exact individual fasting duration, within-visit sampling intervals, collection dates and season were not retained in the analytical extract; participant-level influences of fasting duration or sampling time therefore could not be evaluated.

Serum collection, storage and inductively coupled plasma mass spectrometry (ICP-MS) procedures have been described previously [26,27]. Serum concentrations were treated as time-specific biomonitoring measurements. Their interpretation depends on the element, chemical species and host physiology; for essential elements they are not direct measures of external exposure intensity or toxicity.

Briefly, the procedure used refrigerated centrifugation, continuously monitored −80 °C storage, a 500 µL serum aliquot and 1:10 dilution for duplicate NexION 350 ICP-MS measurements (PerkinElmer, Waltham, MA, USA). Daily five-point calibration used Y-89, Rh-103 and Lu-175 internal standards; quality control included chemical blanks, internal controls and certified reference materials. For each channel, the limit of quantification (LOQ) was derived from 20 reagent blanks [27]. The channel-specific values used in this analysis are reported with units in Table S5. Historical within-/between-run precision, analyte-specific recovery and raw replicate records were unavailable, so numerical assay precision validation could not be provided.

The analytical dataset contained 20 channels representing 19 elements: Li-7, Be-9, Cd-111, As-75, Cr-52, Co-59, Cu-63, Fe-56, Fe-57, Hg-202, Mn-55, Mo-98, Ni-60, Pb-208, Sb-121, Se-78, Sr-88, Tl-205, V-51 and Zn-66. Fe-56 was the primary iron channel; Fe-57 was a duplicate isotope measurement of iron used to assess analytical consistency and was kept outside the primary multiplicity families. Values below LOQ were treated as left-censored.

The parent workflow completed unperformed assays only for elements with ≤5% assay nonperformance among questionnaire responders; Hg and Ni analyses therefore used assay-performed participants, whereas unperformed Zn assays were completed. QC-high records exceeding element-specific upper bounds were set to missing, excluded from imputation-model fitting and donor pools and completed under that workflow. No imputation model was refitted.

We examined Hg/Ni assay availability by environmental pressure area and municipal cluster (Table S16). These comparisons describe observed selection but do not identify the operational reasons for assay nonperformance, which could not be reconstructed. Assay availability weighting addresses only the observed covariates in its models and cannot eliminate selection related to unrecorded laboratory batch, calendar time, specimen availability or other factors.

Interpretation used four predefined classes: toxicological priority (As, Cd, Cr, Sb, Tl, V), measurement-limited (Be, Hg, Ni, Pb), essential/homeostatic (Co, Cu, Fe, Mn, Mo, Se, Zn) and other (Li, Sr). The inherited serum panel is not interchangeable with whole-blood lead or urinary cadmium, the usual matrices for those biomonitoring purposes [30,31]. Speciation was unavailable for arsenic, chromium, mercury, selenium and antimony; isotope labels do not identify chemical species [32,33]. Cross-matrix literature comparisons are therefore qualitative comparisons of direction and plausibility, not numerical calibration of dose, effect magnitude or clinical thresholds.

### 2.3. Laboratory Outcomes

The co-primary laboratory outcomes were NLR and LDL-C. NLR was calculated as absolute neutrophils divided by absolute lymphocytes. LDL-C was measured directly by the study laboratory and analysed as reported, without recalculation. SII, calculated as platelet count (10^3^/µL) multiplied by neutrophils and divided by lymphocytes, was the key secondary outcome. PLR, RDW, serum glucose and homocysteine were secondary or exploratory outcomes.

The blood count outcomes were selected from indices calculable using the available absolute counts. NLR was co-primary, SII added platelet count weighting, and PLR provided a supporting platelet-to-lymphocyte ratio. RDW was a complementary measure of variation in red cell size. These indices do not identify specific inflammatory mechanisms. Infection, inflammation, smoking, obesity, physiological stress and treatment can influence the underlying blood cell counts. CRP/hs-CRP, erythrocyte sedimentation rate and fibrinogen were unavailable. Total leukocyte and monocyte counts were recorded in the parent database but were not included in the retained analytical extract or the specified outcome hierarchy; additional indices requiring these counts were not analysed. Albumin, ferritin, protein fractions, eosinophils and basophils were not principal outcomes because of their broader determinants and to retain a limited outcome hierarchy.

Self-reported diagnosis-linked questions asked about current medication for high blood pressure and cholesterol, as well as insulin or other diabetes medication (counts in Section 3.1 and Table S18); missing responses did not establish non-use. The sensitivity analysis excluded the 66 identified current cholesterol medication users, not all potentially relevant treatments.

NLR, SII, PLR, LDL-C, glucose and homocysteine were natural-log-transformed for regression; RDW was also analysed on the log scale in the main model and in native units as a sensitivity analysis. NLR, SII and PLR were derived separately within each completed dataset from completed blood cell counts, never from pooled component means.

### 2.4. Covariates

Estimands were covariate-adjusted cross-sectional associations, not identified total or direct causal effects. Figure S9 illustrates forward and reverse temporal orderings, shared causes and disease-history selection. Diet, body mass index (BMI), renal function and inflammatory physiology may play different roles under these assumptions; the diagrams neither determined the original adjustment set nor represent fully measured covariate lists.

Model A included age as a restricted cubic spline, sex and 21 municipal-cluster fixed effects. Model B added BMI as a restricted cubic spline, smoking, alcohol, physical activity, socioeconomic deprivation (parent-study score 0–100; higher values indicate greater deprivation), self-reported occupational chemical-hazard exposure and energy intake; alcohol, activity and energy used log(1 + x). Primary Model C added prespecified dietary sources: fish and shellfish for Hg; fish, shellfish and rice for As; dietary iron and calcium for Cd and Pb; dietary iron for Fe; dietary zinc for Zn. The Italian EPIC food-frequency questionnaire assessed habitual diet over the preceding year. Dietary-source quantities used log(1 + intake); fish and shellfish entered separately in g/day, retaining energy and socioeconomic adjustment. Fish species, provenance, preparation and recent intake were unavailable, as were separate validation coefficients for aggregated fish and shellfish variables. Validated dietary Se and Cu estimates and comprehensive supplement use, dose and duration were unavailable. Covariates were not selected using laboratory-outcome *p* values.

### 2.5. Missing Data and Statistical Analysis

Primary analyses used 30 completed datasets generated under the parent SPES censoring-aware multiple-imputation workflow. The procedure used log-Tobit, fixed Box-Cox Tobit or interval-censored Gamma models according to element family and predictive mean matching for routine laboratory variables [34,35,36,37,38,39,40,41,42,43,44]. The imputation models were not refitted for this secondary analysis.

The parent workflow used a broad auxiliary laboratory predictor pool with target-specific selection. LDL-C appeared in the initial recorded predictor selections for the Hg, Se, Cu and Zn censoring models; this check did not establish retention at every subsequent fitting. The inherited imputations were not designed specifically for the present log-outcome and derived-ratio models, and incompatibility could attenuate or otherwise distort associations. The directly quantified and observed CBC sensitivities assess reliance on different completed data components but do not establish substantive-model compatibility.

Concentrations were in µg/L (Fe and Zn: mg/L). The fitted predictor was log concentration divided by the fixed element-specific scale s, the median of 30 primary-cohort log-IQRs. Principal displays express the equivalent per-doubling estimate: log-linear coefficients and confidence limits were multiplied by log(2)/s before exponentiation, leaving *p* and *q* values unchanged. This common relative increment does not represent equivalent absolute dose or comparative potency. Table 2 retains log-IQR estimates and approximate Q1–Q3 intervals in secondary columns; complete results and intervals appear in Tables S6–S10 and S19. At ≥25% left-censoring, Q1 is labelled below LOQ, not an observed quartile. Binary quantification-status and spline contrasts retain their labelled scales. Separate single-element regressions within each completed dataset used municipality-clustered HC1 covariance matrices [45]. Rubin pooling combined coefficients and robust variances with Barnard–Rubin degrees of freedom and cluster-based complete-data degrees of freedom (G−1) [37,38]. Exponentiated estimates express percentage outcome differences.

Model-standardized geometric means were calculated from the existing Model C fits to illustrate association magnitude in original outcome units (SM6; Table S23). These summaries average the model linear predictors over the relevant cohort before exponentiation and pooling across completed datasets. They describe conditional cohort-level associations, not individual effects, causal interventions or biological thresholds.

Benjamini–Hochberg FDR correction [46] was applied to LDL-C and NLR separately (19 element tests each), SII (19), PLR/RDW jointly (38), glucose (19) and exploratory homocysteine (19). The co-primary outcome-specific families supported endpoint-specific inference; no either-endpoint combined success criterion or cross-domain claim was applied. An additional revision-stage BH calculation combined the 38 existing co-primary Model C *p* values; this assessment did not alter the models or original *p* values (Table S24). The linear-model *p* values were two-sided; *q* < 0.05 defined significance within each stated family, without study-wide error control. Secondary restricted cubic spline analyses [47] used four separate 17-test BH families: global association and nonlinearity for LDL-C, as well as global association and nonlinearity for NLR; Be and Pb were excluded because of their predominantly model-generated continuous lower tails. Fe-57 consistency analyses remained outside the FDR families. Element correlations were summarized by median Spearman correlations across imputations (Table S15; Figure S2).

The original sensitivity programme was specified before association modelling, used nominal inference and assessed observed CBC outcomes, quantification status, directly quantified concentrations, cohort masks, omission of BMI or deprivation, renal adjustment, occupational exposure and current smoker exclusions, municipality fixed effects, outcome-specific disease restrictions, QC-high exclusions, native units, Hg/Ni assay availability weighting and alternative interval-censored Gamma completion (Table S1). CR2 and influence diagnostics used predefined triggers; influence sensitivity meant direction reversal or a >25% relative change in effect magnitude. Post-primary exploratory additions, also nominal, comprised joint Se/Cu/Zn–LDL-C modelling, exclusion of 66 identified current cholesterol medication users and extension of Hg/Ni weighting to Ni–homocysteine. Sensitivities were interpreted by magnitude, direction and uncertainty, not independent validation. Table S2 distinguishes specification, execution and descriptive re-expression. LDL-C was the sole lipid outcome modelled; the study was not externally preregistered. Association analyses used R 4.6.1 on Windows 11; model-standardized geometric means were calculated in R 4.5.1. Package versions and reproducibility details are provided in Supplementary Methods SM10.

Single-element models estimated conditional associations, not an overall mixture effect. Correlation may confound these associations; heterogeneous measurement support does not remove the value of mixture analysis. The nominal, post-primary joint Se/Cu/Zn–LDL-C sensitivity did not identify independent causal effects. Dietary zinc adjustment differed from individual Se and Cu models, preventing attribution of estimate differences solely to mutual element adjustment.

### 2.6. Ethics

The parent SPES programme was approved by the Ethics Committee of IRCCS Fondazione G. Pascale (Extraordinary Commissioner’s Resolution No. 590, 3 August 2016). The present secondary analysis used a de-identified analytical extract under the parent study’s data-governance framework and involved no new participant contact, specimen collection or laboratory testing.

## 3. Results

### 3.1. Cohort and Measurement Support

The primary cohort included 4026 participants, with a median age of 35 years (IQR 27–43; range 19–55); 76 (1.9%) were outside the stated recruitment age range of 20–49. There were 2165 women (53.8%); median BMI was 24.62 kg/m^2^ and 1246 participants (30.9%) currently smoked. Median LDL-C was 116 mg/dL, median NLR was 1.60, and median SII was 363.9.

A physical examination was recorded as performed for all 4026 participants. General appearance was normal in 3980 of the 4025 participants with that assessment recorded (98.9%). Among 4013 participants with all 11 examination domains assessed, 3932 (98.0%) had no clinically significant abnormality in any domain.

Original measurement availability differed from the completed-data model denominators. LDL-C was observed in all 4026 participants; the observed-data counts were 3943 for NLR and RDW and 3941 for SII and PLR, requiring the relevant constituent counts for each index. Original observed and missing/unknown counts and imputation eligibility are reported by variable in Table 1 and Table S4. Three participants had no usable BMI after the original data-preparation rules, leaving 4023 in most fully adjusted models. The Hg scaling set included 3259 assay-performed primary-cohort participants; one also lacked BMI, leaving *n* = 3258 for Model C. Ni had *n* = 2213 for both scaling and Model C.

Of 444 participants reporting physician-diagnosed hypertension, 180 reported current medication for high blood pressure. Of 580 reporting high cholesterol or triglycerides, 66 reported current cholesterol medication. Of 32 reporting diabetes, 22 reported insulin or another diabetes medication (Supplementary Table S18). These counts describe users identified by the diagnosis-linked items and do not establish complete ascertainment of current treatment in the cohort.

In the full 4188-participant analytical cohort, 785 Hg assays (18.7%), 1861 Ni assays (44.4%) and 180 Zn assays (4.3%) were unperformed. Among performed assays, left-censoring was greatest for Pb (71.0%), Be (69.2%), Cd (44.7%), Cr (41.1%), Sb (36.2%), Ni (30.5%), Tl (25.7%) and Hg (25.5%). Hg/Ni assay availability differed by environmental pressure area and municipal cluster (Table S16). The unperformed Zn assays met the ≤5% rule for completion; the directly quantified Zn analysis provided an observed-data sensitivity check. Across the complete 20-channel panel, 37 records flagged as exceeding quality-control upper bounds (QC-high) were set to missing and completed. Baseline and measurement-support details are shown in Table 1 and Supplementary Tables S3–S5.

### 3.2. Primary Outcomes

**Figure 1:**
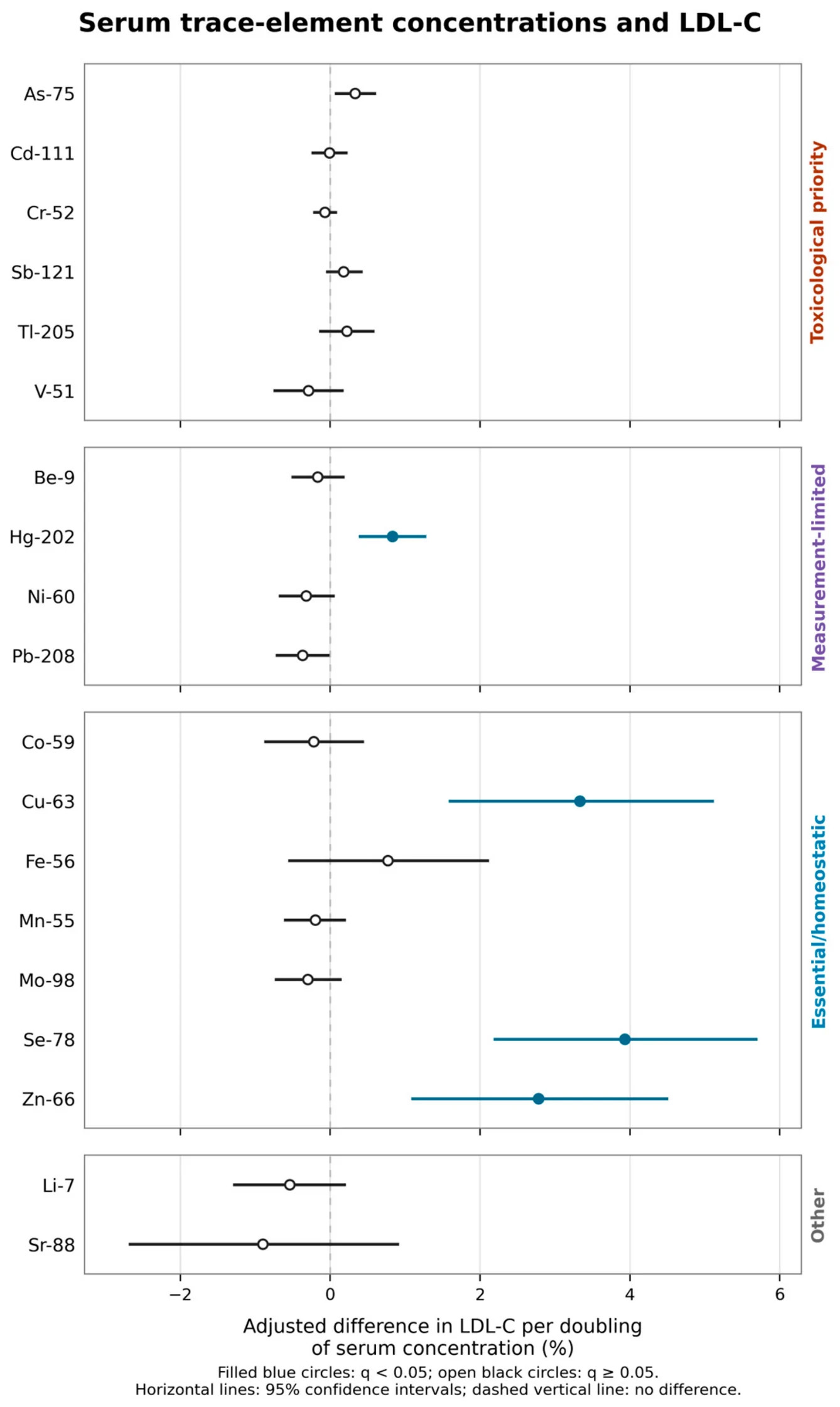
Serum trace-element concentrations and LDL-C. Points show the adjusted percentage difference in LDL-C per doubling of serum element concentration in Model C; horizontal lines show 95% confidence intervals. The dashed vertical line indicates no difference. Filled blue circles indicate *q* < 0.05 and open black circles *q* ≥ 0.05 within the original 19-test LDL-C family. Panels show the predefined interpretive classes. Doubling is a common relative concentration increment, not a common absolute dose or a measure of comparative biological potency. Log-IQR estimates and their element-specific concentration contrasts are retained in Tables S7 and S19.

Four serum elements were positively associated with LDL-C after the original outcome-specific FDR correction: Se, Cu, Zn and Hg. Per doubling of concentration, the estimated differences were +3.93% (95% CI 2.18–5.70), +3.33% (1.58–5.12), +2.78% (1.08–4.51) and +0.83% (0.38–1.28), respectively (Table 2; Figure 1). All four also met *q* < 0.05 in the revision-stage BH assessment across the 38 co-primary comparisons (Table S24). For the original element-specific log-IQR increments, the corresponding relative estimates were +2.04%, +1.55%, +1.26% and +3.72%; separate native-outcome sensitivity models estimated +2.55, +1.81, +1.41 and +4.35 mg/dL over those same increments (Table S13). These modest cross-sectional differences do not estimate treatment benefit. The joint Se/Cu/Zn model and the Hg quantification-status analysis qualify these findings, as described below. Corresponding LDL-C spline curves are shown in Figures S3–S6.

**Figure 2:**
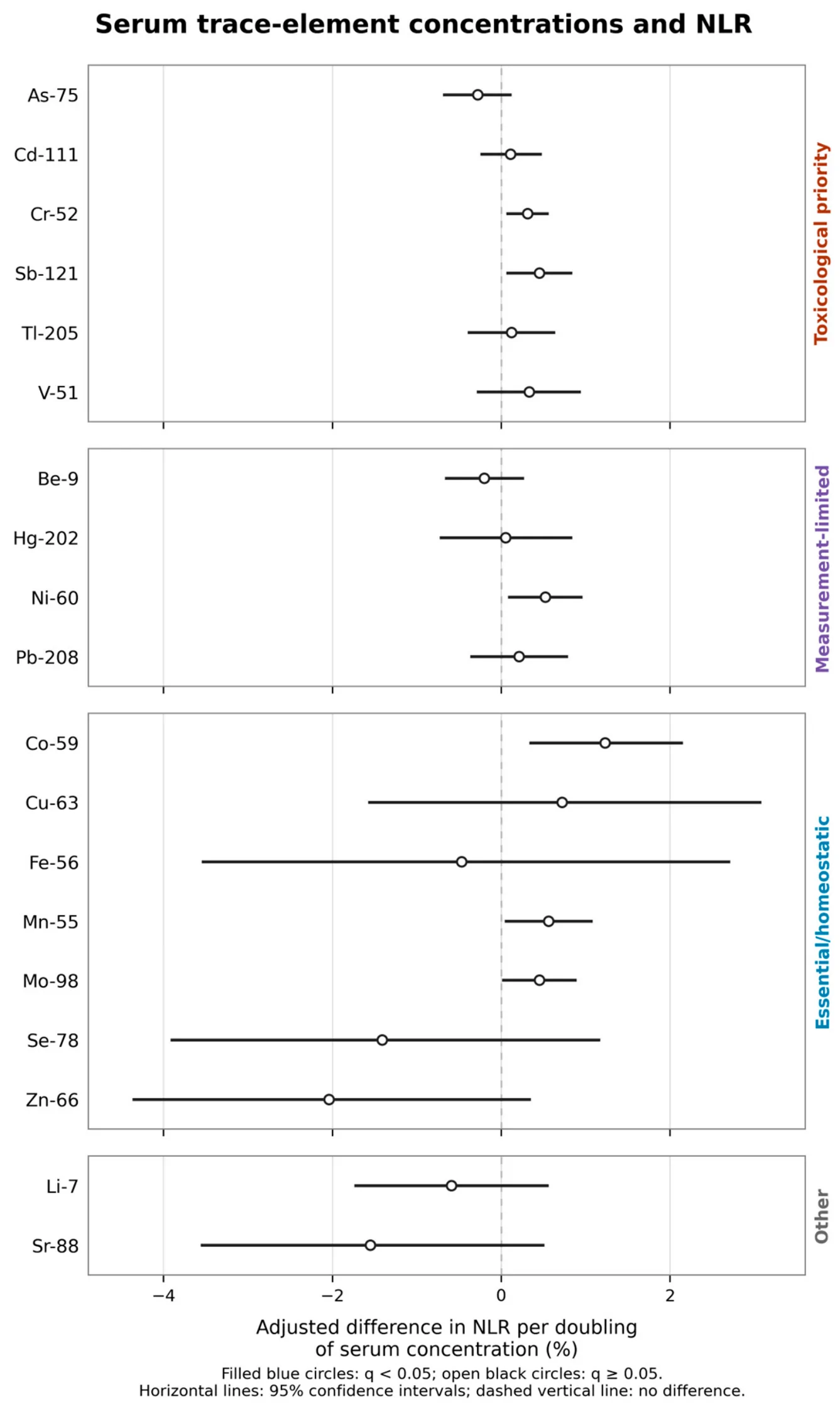
Serum trace-element concentrations and NLR. Points show the adjusted percentage difference in NLR per doubling of serum element concentration in Model C; horizontal lines show 95% confidence intervals. The dashed vertical line indicates no difference. All circles are open because no linear association met *q* < 0.05 within the original 19-test NLR family. Doubling is a common relative concentration increment, not a common absolute dose or a measure of comparative biological potency. Log-IQR estimates and their element-specific concentration contrasts are retained in Tables S6 and S19. The separate secondary global spline association for Co does not replace the primary linear result.

No primary linear element–NLR association met the original outcome-specific FDR threshold, and none met the revision-stage BH threshold across the 38 co-primary comparisons (Figure 2; Tables S6 and S24). This non-rejection does not establish absence of association; interpretation depends on the confidence intervals, censoring and measurement precision. In secondary spline analysis, Co had an FDR-significant global association, but the specifically nonlinear component was not significant (Table S12; Figure S7). The global spline result does not supersede the primary linear analysis.

### 3.3. Secondary and Exploratory Outcomes

**Figure 3:**
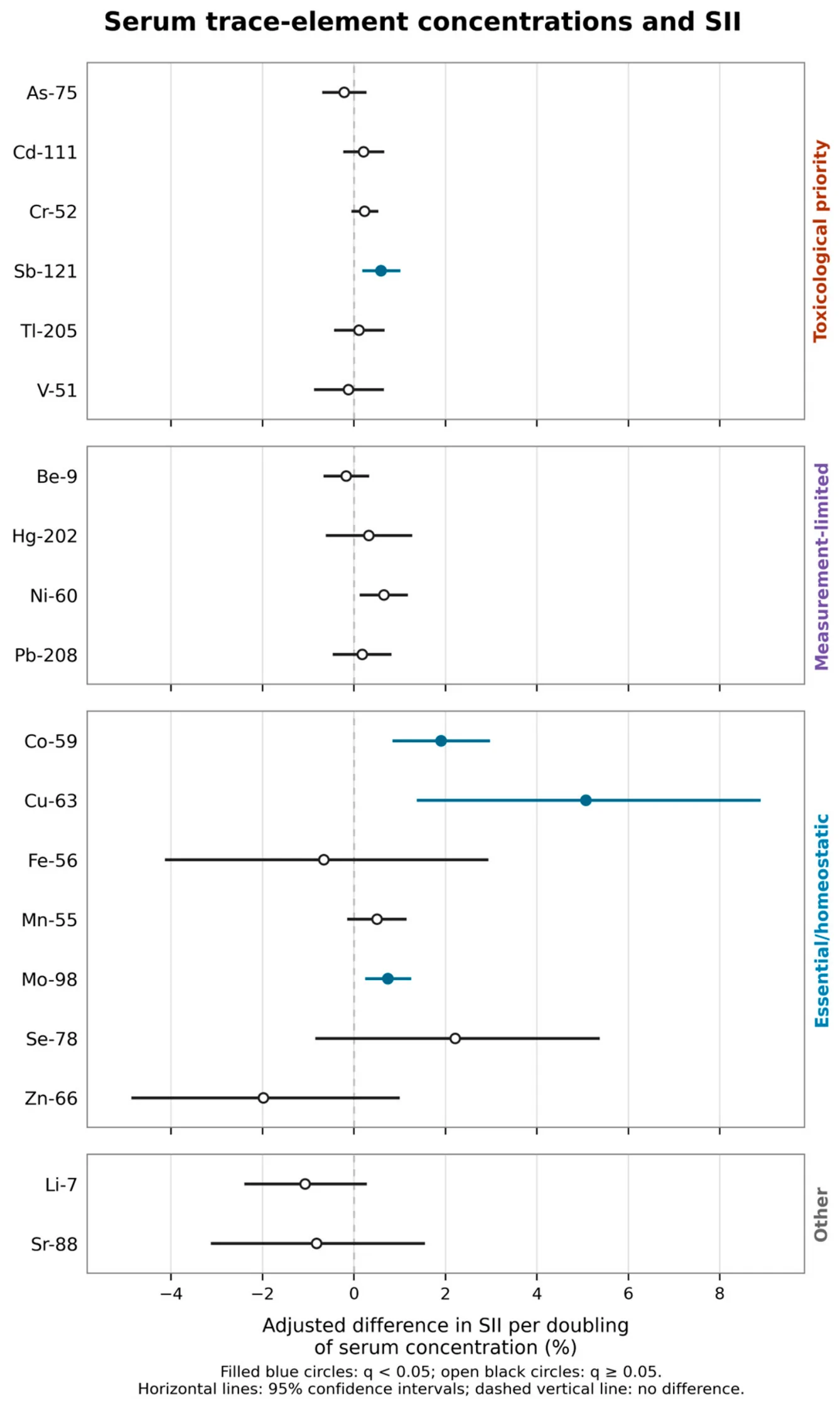
Serum trace-element concentrations and SII. Points show the adjusted percentage difference in SII per doubling of serum element concentration in Model C; horizontal lines show 95% confidence intervals. The dashed vertical line indicates no difference. Filled blue circles indicate *q* < 0.05 and open black circles *q* ≥ 0.05 within the 19-test SII family. Doubling is a common relative concentration increment, not a common absolute dose or a measure of comparative biological potency. Log-IQR estimates and their element-specific concentration contrasts are retained in Tables S8 and S19.

Per original element-specific log-IQR, SII was positively associated with Co-59 (+1.46%; 95% CI 0.65–2.28; *q* = 0.009), Mo-98 (+1.39%; 0.44–2.35; *q* = 0.032), Cu-63 (+2.34%; 0.64–4.07; *q* = 0.034) and Sb-121 (+4.29%; 1.29–7.38; *q* = 0.032) (Figure 3). On a two-fold concentration scale, the estimates were +1.90%, +0.74%, +5.07% and +0.59%, respectively. No PLR association survived FDR correction.

Per original element-specific log-IQR, Fe-56 was associated with lower RDW (−1.35%; 95% CI −1.82–−0.87; *q* < 0.001). Fe-57 yielded a concordant estimate (−1.20%; *p* < 0.001), supporting channel consistency (Table S11). No glucose association survived FDR correction. On the same log-IQR scale, exploratory analyses showed that Ni-60 (+3.36%; 1.63–5.12; *q* = 0.003) and Mo-98 (+0.77%; 0.32–1.22; *q* = 0.009) were associated with higher homocysteine (Table 2). Their two-fold estimates were +0.47% and +0.41%, respectively. The cross-outcome pattern is summarized in Figure S1.

### 3.4. Absolute Magnitude of the Associations

The LDL-C differences were modest in original measurement units: for example, the illustrative Se contrast of approximately 161–232 µg/L corresponded to model-standardized geometric means of 113.2 and 115.5 mg/dL, a difference of 2.3 mg/dL. These cohort-level conditional summaries, reported with uncertainty in Table S23, are distinct from the native-outcome regression estimates in Table S13 and do not describe individual responses, causal thresholds or treatment targets.

### 3.5. Sensitivity Analyses and Imputation Precision

The four primary LDL-C associations retained positive estimates across the reported cohort, covariate, covariance, geographical and medication-exclusion sensitivity analyses (Table 3; Tables S13 and S21). For Hg, the observed quantifiable-versus-below-LOQ contrast was +4.08% LDL-C (95% CI 1.77–6.43; *n* = 3253), whereas the continuous estimate among directly quantified concentrations was +1.89% per original fixed log-IQR (−1.46–5.36; *n* = 2416; nominal *p* = 0.269). The latter estimate did not demonstrate a clear gradient and remains compatible with a range of associations. The quantification-status comparison uses observed status and observed LDL-C without assigning numerical Hg concentrations below the LOQ; it does not validate the primary continuous concentration model. The contrasts and sample restrictions differ, and the LOQ is an analytical boundary, not a biological threshold. Assay availability weighting yielded +3.63% on the same log-IQR scale (1.61–5.70), close to the primary +3.72% estimate. This stability provides reassurance regarding sensitivity to reweighting on measured covariates, while unmeasured selection bias remains possible. Influence diagnostics did not reverse any of the four LDL-C estimates; Cu and Zn exceeded the predefined sensitivity threshold because trimming or robust regression increased their estimates (Table S17).

In the post-primary exploratory joint Se/Cu/Zn model (*n* = 4023), the per-doubling LDL-C estimates were +2.99% for Se (95% CI 0.89–5.13), +1.88% for Cu (−0.33–4.14) and +0.24% for Zn (−2.09–2.63) (Table 2; Table S20). Se retained a positive conditional association, whereas the Cu and Zn confidence intervals included zero. The joint model included dietary zinc, which was absent from the individual Se and Cu models; changes in estimates therefore cannot be attributed solely to mutual adjustment for serum elements. This analysis used nominal inference and does not estimate independent causal effects or an overall mixture effect.

SII sensitivity estimates, expressed per original element-specific log-IQR, varied across restrictions. For Sb, the directly quantified estimate was +0.93% (95% CI −8.50–11.33) and the strict-cohort estimate was +3.33% (−0.34–7.14). The strict-cohort Cu estimate was +1.48% (−0.40–3.38), and the directly quantified Co estimate was +0.78% (−0.81–2.39). Mo remained positive under both restrictions. The alternative Family-C imputation set yielded similar estimates. For exploratory Ni–homocysteine, assay availability weighting yielded +3.34% per original fixed log-IQR (1.44–5.28), with effective *n* = 1991 and stabilized weights of 0.56–2.28 (Table S22). The estimate was close to the primary +3.36%, indicating little sensitivity to reweighting on measured covariates; unrecorded determinants of assay availability remain unaddressed. All sensitivity comparisons use nominal inference.

Within the two primary outcome families, the maximum fraction of missing information was 0.142, minimum relative efficiency was approximately 99.5%, and maximum Monte Carlo error-to-total-standard-error ratio was 0.065 (Table S14). Across all Model C analyses, the maximum fraction of missing information was 0.177. No coefficient triggered a high-FMI or high-Monte-Carlo-error flag. These measures describe precision conditional on the inherited imputation procedure; they do not establish compatibility with every substantive model or absence of imputation-related bias.

## 4. Discussion

In this secondary cross-sectional analysis of 4026 community residents, serum Se, Cu, Zn and Hg were positively associated with LDL-C, with modest differences of approximately 1–4 mg/dL in separate native-outcome models over the original element-specific log-IQR increments. Se retained a positive conditional association in the joint model, whereas Cu and Zn attenuated under its adjustment specification. LDL-C differed by Hg quantification status, without a clearly demonstrated gradient among directly quantified Hg concentrations. No primary linear NLR association met the FDR threshold, and the SII findings varied across sensitivity analyses. These findings describe concentration–marker associations and do not establish disease risk or causal effects.

The Hg result requires interpretation by measurement contrast. The quantifiable-versus-below-LOQ comparison was positive, whereas the continuous estimate among directly quantified concentrations was imprecise and did not demonstrate a clear gradient. A quantification-status comparison avoids assigning numerical Hg values below the LOQ but remains subject to residual confounding and assay-selection bias. Published blood-Hg studies reported positive lipid associations in different populations [48,49,50]; differences in matrix, exposure range and confounding control limit direct comparison. Fish and shellfish intake were included in Model C, but quantifiable Hg may still reflect dietary patterns and correlated exposures. The LOQ should not be interpreted as a threshold of biological harm.

The positive Se–LDL-C association is consistent in direction with previous population studies. Laclaustra et al. reported an adjusted LDL-C difference of 12.7 mg/dL (95% CI 3.3–22.2) between the highest and lowest serum Se quartiles in NHANES 2003–2004 [22], and Bleys et al. reported a corresponding difference of 10.9 mg/dL (6.4–15.4) in NHANES III [51]. Higher plasma Se was also associated with higher non-HDL cholesterol in British adults [52]. In SPES, the illustrative fixed Se contrast of approximately 161–232 µg/L corresponded to a standardized geometric-mean LDL-C difference of 2.3 mg/dL (Table S23). Although these studies agree in direction, their different populations, concentration contrasts and outcome models do not permit direct comparison of magnitude. LDL-C was calculated in the NHANES 2003–2004 report but directly measured in SPES. The copper comparison is less direct. In NHANES 2011–2014, Song et al. reported 8.42 mg/dL higher total cholesterol in the highest than the lowest Cu tertile; the significant LDL-specific finding was higher odds of elevated LDL-C in the middle versus the lowest tertile (odds ratio 1.48; 95% CI 1.03–2.14) [23]. These observations provide context for the SPES Cu association, whose attenuation in the exploratory joint model also indicates dependence on the adjustment specification.

Randomized evidence addresses whether supplementation changes lipid concentrations. In the main analysis of the Danish PRECISE trial, five years of selenium supplementation did not significantly change the reported cholesterol measures relative to placebo [53]. AREDS Report No. 7 similarly found increased serum zinc without significant lipid changes after five years of a formulation containing zinc together with copper [54]. Because that formulation contained both elements, it does not isolate the effect of either zinc or copper. Naturally occurring serum concentration differences and supplementation are distinct comparisons; our findings do not justify selenium, copper or zinc restriction or supplementation for lipid management.

Serum Se, Cu and Zn vary with nutrient intake, hepatic synthesis, transport proteins and inflammatory redistribution [11,12,13,14,15]. Lipid metabolism or hepatic physiology may therefore alter circulating element concentrations, while shared determinants may influence both elements and LDL-C. The cross-sectional joint model cannot distinguish these directions. Albumin and total protein were available in SPES, but SELENOP, GPX3, ceruloplasmin, carrier-bound metal fractions, C-reactive protein and chemical speciation were not measured. The available data therefore leave transport and redistribution as plausible explanations rather than demonstrated mechanisms.

Pb and Cd did not meet the LDL-C FDR threshold, and the nominal As estimate also did not meet it. This does not contradict prospective studies of cardiovascular outcomes [16,17,18,19,20]: the outcomes, exposure windows, populations and matrices differ. A single serum measurement, especially when extensively censored or poorly suited to longer-term exposure assessment, cannot establish absence of chronic toxicity.

No primary linear element–NLR association met the FDR threshold. This does not exclude smaller associations or associations obscured by censoring, measurement error or restricted concentration ranges. The secondary global Co spline association, without a significant specifically nonlinear component, requires replication. NLR and SII have been associated with incident disease in other studies [3,4,5,6,7], but remain nonspecific blood count indices and are not validated disease-risk surrogates in this analysis.

The secondary SII findings were less consistent across sensitivity analyses. Cu attenuated in the strict cohort, Co and Sb had imprecise continuous estimates among directly quantified concentrations, and SII was higher for quantifiable than for below-LOQ Sb. Mo remained positive under both restrictions. In NHANES, Zhong et al. reported positive associations of blood Cd and Mn with SII [24]. The corresponding SPES estimates were also positive but did not meet the FDR threshold, so the results differ in statistical evidence rather than direction. Serum versus whole-blood measurements, concentration distributions and covariate control may contribute to differences between studies, without establishing their cause. Sun et al. also reported positive metal-mixture associations with SII, although statistical evidence differed between the weighted quantile sum and quantile g-computation models [55]. These mixture estimates answer a different question from the individual-element estimates reported here. SII incorporates platelet count but does not measure platelet activity, and inflammation can itself redistribute circulating elements [14,15]. An older Italian population with selenium deficiency had higher inflammatory markers, including NLR and C-reactive protein [56]; its findings concern a different nutritional context from the higher serum selenium range in SPES.

The inverse Fe–RDW association, concordant across Fe-56 and Fe-57, was consistent with iron availability and erythropoietic physiology. For homocysteine, the exploratory Ni and Mo findings can be compared with distinct human studies. Katko et al. reported an inverse univariate correlation between serum Ni and total homocysteine in 122 haemodialysis patients (r = −0.289), opposite in direction to SPES [57]. Renal disease, treatment and the difference between an unadjusted correlation and an adjusted community-cohort estimate limit that comparison. In contrast, Long et al. found positive plasma Mo–homocysteine associations in 2989 Dongfeng–Tongji participants in both single- and multiple-metal models [58], consistent in direction with SPES. Our standardized geometric-mean differences were small: 0.06 µmol/L for the fixed Mo log-IQR contrast and 0.21 µmol/L for the Ni quantification-status contrast (Table S23). These are different estimands and should not be compared as relative potency. The positive continuous Ni estimate was similar after assay availability weighting, providing reassurance about sensitivity to measured covariates while leaving unrecorded selection mechanisms unresolved.

The contribution of this study is to characterize modest serum concentration–marker associations in community biomonitoring. These estimates provide observational evidence for comparison across populations and for prospective investigation. They do not quantify population-attributable disease burden or establish benefit from lowering or increasing any circulating element concentration.

Prospective studies with repeated element and outcome measurements could clarify temporal ordering and within-person variability. Priorities include validated dietary and supplement assessment, current medication characterization, inflammatory and hepatic biomarkers, standard exposure matrices, chemical speciation and documented analytical batches. Mixture methods should address a defined joint-exposure question with explicit treatment of measurement limitations. Genetic instrumental-variable approaches may inform selected essential-element questions if suitable instruments are available and their validity and pleiotropy are carefully assessed.

Strengths include the community-based cohort, broad serum element panel, outcome hierarchy specified before association modelling, clustered inference, explicit multiplicity control and complementary sensitivity analyses.

These findings should be interpreted in light of the study’s design and measurement constraints. The cross-sectional data cannot establish whether element concentrations preceded the biomarker differences, resulted from them or shared other determinants. A single serum measurement may not represent long-term exposure or usual nutrient status. Serum is not the preferred matrix for several elements, and chemical speciation was unavailable, limiting comparison with studies using blood, urine or species-specific measurements. Historical numerical precision and recovery summaries were also unavailable, although the published laboratory workflow describes quality-control procedures.

Diet, supplements, treatment and unrecognized disease may contribute to residual confounding. Diagnosis-linked questionnaire items identified self-reported current medication for high blood pressure, cholesterol and diabetes, but did not provide complete current drug-class ascertainment. Physical examination before blood collection and the self-administered questionnaire characterize the available clinical assessment; they could not systematically exclude infection, inflammatory or haematological disease, recent surgery or subclinical conditions affecting the nonspecific NLR and SII indices. Although the published procedure specified an instruction to fast for at least eight hours and a staff check, individual fasting duration and sampling time were unavailable in the analytical extract.

The composition of the analytical cohort also limits interpretation. Invitation/contact and nonparticipant data needed to estimate participation and assess nonresponse systematically were not collected. Parent recruitment eligibility included a negative reported HCV/HBV/HIV history, which preceded the present analytical restriction and did not establish absence of all infection. Restriction to participants without the specified self-reported histories of physician-diagnosed tumour, myocardial infarction, angina or stroke may introduce selection or survivor bias. Hg and Ni analyses were further restricted by assay availability, and the historical operational reasons for nonperformance could not be reconstructed. Similar weighted and primary estimates address sensitivity to measured covariates but cannot exclude selection related to unrecorded laboratory batch, calendar time, specimen availability or other factors.

Interpretation also depends on the inherited imputation models, whose equations were not constructed to guarantee compatibility with every outcome and substantive-model term. Such incompatibility may attenuate or otherwise distort associations, particularly when imputation is extensive. Small Monte Carlo error and high relative efficiency describe precision conditional on that procedure rather than its compatibility with the analysis model. The sensitivity analyses therefore qualify the primary results; their nominal inference provides neither independent validation nor additional multiplicity-controlled discoveries.

## 5. Conclusions

In this secondary cross-sectional SPES analysis, the clearest findings were modest serum concentration–LDL-C associations, corresponding to differences of approximately 1–4 mg/dL per element-specific log-IQR in separate native-outcome models. Se retained a positive conditional association in the joint model, Cu and Zn attenuated under its adjustment specification, and LDL-C differed by Hg quantification status without a clearly demonstrated gradient among quantified Hg concentrations. No primary linear NLR association met the multiplicity-adjusted threshold. Prospective studies with repeated measurements are needed to establish temporal relationships and assess whether these biomarker patterns predict subsequent outcomes. Any role for modifying element intake would require evidence from studies specifically designed to evaluate the benefits and harms of intervention.

## Figures and Tables

**Table 1:** Characteristics of the primary cohort (*N* = 4026).

Characteristic	Value	Observed/Known *n*	Missing/Unknown *n*	Imputation Status
Age, years	35 [27–43]	4026	0	No
Women	2165 (53.8%)	4026	0	No
Men	1861 (46.2%)	4026	0	No
Body mass index, kg/m^2^	24.62 [22.23–27.68]	4023	3	No; residual model exclusion
Never/former/current smoker	2050/730/1246	4026	0	No
Alcohol intake, g/day	1.25 [0.15–7.11]	4026	0	No
Physical activity, MET-h/week	36.00 [15.75–66.75]	4026	0	No
Socioeconomic deprivation score	29.20 [8.30–37.50]	4026	0	No
Occupational exposure, yes	802 (19.9%)	4026	0	No
Creatinine, mg/dL (observed)	0.80 [0.70–0.90]	4026	0	No
Directly measured LDL-C, mg/dL (observed)	116 [95–140]	4026	0	No
NLR (observed)	1.60 [1.24–2.05]	3943	83	Derived after constituent-count imputation
SII (observed)	363.9 [269.8–493.5]	3941	85	Derived after constituent-count imputation
RDW, % (observed)	12.04 [11.61–12.60]	3943	83	Predictive mean matching
Glucose, mg/dL (observed)	77 [72–83]	4026	0	No
Homocysteine, µmol/L (observed)	8.10 [7.20–9.35]	4026	0	No
Reported physician-diagnosed hypertension	444 (11.0%)	4026	0	No
Current high-blood-pressure medication among those diagnosed	180/444 (40.5%)	444	0	No; denominator 444
Reported high cholesterol or triglycerides	580 (14.4%)	4026	0	No
Current cholesterol medication among those diagnosed	66/580 (11.4%)	580	0	No; denominator 580
Reported diabetes	32 (0.8%)	4026	0	No
Insulin or other diabetes medication among those diagnosed	22/32 (68.8%)	32	0	No; denominator 32

Values are median [IQR], *n* (%) or *n*/*N* (%). Observed/known and missing/unknown counts refer to original primary-cohort records (*N* = 4026); BMI counts refer to availability for the primary models after the original data preparation. For NLR, SII and PLR, availability requires every constituent count used in that index. Descriptive laboratory summaries use observed measurements. RDW and the constituent blood counts were eligible for predictive-mean-matching imputation; the indices were derived after component completion. Completed-data model *n* can differ from observed-data *n* because of imputation, exposure availability and residual covariate missingness. Medication follow-up denominators comprise participants reporting the corresponding diagnosis; blanks outside that stratum do not establish non-use. Element-assay support in Table S5 uses its separately labelled full-cohort denominator of 4188 and distinguishes unperformed assays, below-LOQ measurements and post-analytical plausibility exclusions. NLR, neutrophil-to-lymphocyte ratio; SII, systemic immune–inflammation index; RDW, red cell distribution width; LDL-C, low-density lipoprotein cholesterol. MET, metabolic equivalent of task; IQR, interquartile range.

**Table 2:** Single-element associations and exploratory joint LDL-C model. (**A**) FDR-significant single-element Model C associations. (**B**) Post-primary exploratory joint Se/Cu/Zn–LDL-C analysis.

(**A**)							
**Laboratory Outcome**	**Serum Element**	***n***	**Adjusted Outcome Difference per Doubling, % (95% CI)**	***p***	**Original Outcome-Family *q***	**Approximate Serum Concentration Interval**	**Adjusted Outcome Difference per Original Log-IQR, % (95% CI)**
LDL-C	Se-78	4023	+3.93% (+2.18% to +5.70%)	1.30 × 10^−5^	2.47 × 10^−4^	161–232 µg/L	+2.04% (+1.14% to +2.95%)
LDL-C	Cu-63	4023	+3.33% (+1.58% to +5.12%)	2.14 × 10^−4^	0.002	842–1164 µg/L	+1.55% (+0.74% to +2.36%)
LDL-C	Hg-202	3258	+0.83% (+0.38% to +1.28%)	3.47 × 10^−4^	0.002	<0.119–2.52 µg/L *	+3.72% (+1.69% to +5.79%)
LDL-C	Zn-66	4023	+2.78% (+1.08% to +4.51%)	0.001	0.007	1.22–1.66 mg/L	+1.26% (+0.49% to +2.04%)
SII	Co-59	4023	+1.90% (+0.84% to +2.97%)	4.89 × 10^−4^	0.009	0.712–1.21 µg/L	+1.46% (+0.65% to +2.28%)
SII	Mo-98	4023	+0.74% (+0.24% to +1.25%)	0.004	0.032	0.335–1.23 µg/L	+1.39% (+0.44% to +2.35%)
SII	Sb-121	4023	+0.59% (+0.18% to +1.01%)	0.005	0.032	<0.0020–0.192 µg/L *	+4.29% (+1.29% to +7.38%)
SII	Cu-63	4023	+5.07% (+1.37% to +8.89%)	0.007	0.034	842–1164 µg/L	+2.34% (+0.64% to +4.07%)
RDW	Fe-56	4023	−1.66% (−2.24% to −1.08%)	8.92 × 10^−8^	3.39 × 10^−6^	0.903–1.58 mg/L	−1.35% (−1.82% to −0.87%)
Homocysteine	Ni-60	2213	+0.47% (+0.23% to +0.71%)	1.60 × 10^−4^	0.003	<0.0212–2.69 µg/L *	+3.36% (+1.63% to +5.12%)
Homocysteine	Mo-98	4023	+0.41% (+0.17% to +0.65%)	9.48 × 10^−4^	0.009	0.335–1.23 µg/L	+0.77% (+0.32% to +1.22%)
(**B**)							
**Element**	**Individual Model C *n***	**Individual Estimate per Doubling, % (95% CI)**		**Joint Model *n***	**Joint Estimate per Doubling, % (95% CI)**		**Joint Nominal *p***
Se-78	4023	+3.93 (2.18–5.70)		4023	+2.99 (0.89–5.13)		0.005
Cu-63	4023	+3.33 (1.58–5.12)		4023	+1.88 (−0.33–4.14)		0.095
Zn-66	4023	+2.78 (1.08–4.51)		4023	+0.24 (−2.09–2.63)		0.839

Approximate Q1–Q3 intervals are shown for serum trace-element concentrations in original laboratory units (µg/L, except Fe and Zn in mg/L). * For Hg-202, Ni-60 and Sb-121, Q1 was below the LOQ and is shown as <LOQ; the completed-distribution fold equivalent is reported in Supplementary Table S19. Log-IQR effects represent element-specific population contrasts and are not directly comparable as relative potency across elements. Per-doubling effects use a common two-fold concentration contrast; *p* and *q* values are unchanged by rescaling. Benjamini-Hochberg correction was applied to NLR (19 tests), LDL-C (19), SII (19), the joint PLR/RDW family (38), glucose (19) and exploratory homocysteine (19). Homocysteine was exploratory; Fe-57 consistency estimates were outside FDR families. Complete Model C results are reported in Tables S6–S10. Individual Se and Cu models used the common Model B covariates; the individual Zn model additionally included dietary zinc. The joint model included Se, Cu and Zn simultaneously plus Model B covariates and dietary zinc. Model B comprises age and BMI splines, sex, municipal-cluster fixed effects, smoking, alcohol, physical activity, deprivation, occupational exposure and energy intake, with transformations as specified in Section 2.4. The joint analysis is post-primary exploratory and uses nominal inference. Differences between individual and joint estimates cannot be attributed solely to mutual adjustment because the adjustment sets differ. Original log-IQR estimates are retained in Table S20. CI, confidence interval; IQR, interquartile range.

**Table 3:** Sensitivity analyses of FDR-significant LDL-C associations.

Serum Element	Main Model C	Strict Cohort	No Reported Dyslipidaemia	Excluding Identified Cholesterol Medication Users	Directly Quantified Concentration	Quantified vs. <LOQ
Hg-202	+3.72% (+1.69 to +5.79); *p* = 3.47 × 10^−4^; *q* = 0.002	+2.47% (+0.32 to +4.67); *p* = 0.025	+3.12% (+1.07 to +5.22); *p* = 0.003	+3.49% (+1.51 to +5.51); *p* = 5.74 × 10^−4^	+1.89% (−1.46 to +5.36); *p* = 0.269	+4.08% (+1.77 to +6.43); *p* = 5.49 × 10^−4^
Se-78	+2.04% (+1.14 to +2.95); *p* = 1.30 × 10^−5^; *q* = 2.47 × 10^−4^	+2.08% (+1.08 to +3.08); *p* = 5.09 × 10^−5^	+2.11% (+1.12 to +3.10); *p* = 3.50 × 10^−5^	+2.18% (+1.20 to +3.16); *p* = 1.69 × 10^−5^	+2.37% (+1.46 to +3.28); *p* = 6.16 × 10^−7^	Not informative (1 censored value)
Cu-63	+1.55% (+0.74 to +2.36); *p* = 2.14 × 10^−4^; *q* = 0.002	+1.56% (+0.60 to +2.54); *p* = 0.002	+1.48% (+0.65 to +2.31); *p* = 5.40 × 10^−4^	+1.65% (+0.87 to +2.44); *p* = 4.29 × 10^−5^	+1.68% (+0.70 to +2.68); *p* = 8.55 × 10^−4^	Not informative (1 censored value)
Zn-66	+1.26% (+0.49 to +2.04); *p* = 0.001; *q* = 0.007	+1.13% (+0.34 to +1.92); *p* = 0.005	+1.25% (+0.49 to +2.02); *p* = 0.001	+1.52% (+0.81 to +2.23); *p* = 3.85 × 10^−5^	+1.62% (+0.60 to +2.64); *p* = 0.002	Not informative (1 censored value)

Continuous cells report adjusted percentage differences per trace-element log-IQR; quantified-versus-<LOQ reports the binary contrast between quantified and left-censored serum concentrations. These are distinct estimands. The strict cohort comprised primary-cohort participants with additional known negative histories of hypertension, dyslipidaemia and diabetes (*n* = 3120; Table S3). Primary Model C *q* values are shown in the main column. Sensitivity analyses were not assigned separate FDR families; therefore, sensitivity-specific *q* values were not calculated. Additional denominators, CR2 estimates and sensitivity analyses are reported in Supplementary Table S13.

## Data Availability

De-identified participant-level data and frozen imputation objects are controlled under SPES governance and are not publicly released because of ethics and re-identification constraints. They may be made available to editors and peer reviewers for verification under a confidentiality agreement and prohibition of re-identification. The final analysis specification, analytic code, session information and aggregate outputs are retained by the authors and may be made available for editorial or reproducibility review on reasonable request, subject to study governance. All results needed to evaluate the present report are provided in the main article and Supplementary Materials.

## Supplementary Materials

The following supporting information can be downloaded at: https://www.mdpi.com/article/10.3390/toxics14090824/s1, Supplementary Methods and Results; Table S1: Sensitivity analysis definitions; Table S2: Timing and inferential status of the analysis programme; Table S3: Cohort counts; Table S4: Primary cohort characteristics; Table S5: Element measurement support; Table S6: Complete Model C results for NLR; Table S7: Complete Model C results for LDL-C; Table S8: Complete Model C results for SII; Table S9: Complete Model C results for PLR and RDW; Table S10: Complete Model C results for glucose and homocysteine; Table S11: Fe-57 analytical consistency channel; Table S12: Restricted cubic spline tests; Table S13: Selected robustness and native-unit analyses for FDR-significant or clinically salient findings; Table S14: Highest FMI values within the two primary outcome families; Table S15: Largest median inter-element Spearman correlations; Table S16: Largest standardized differences by Hg/Ni assay availability; Table S17: Influence diagnostics for FDR-significant LDL-C associations; Table S18: Linked parent-questionnaire medication information; Table S19: Element-specific log-IQR increments and original-unit serum concentration contrasts; Table S20: Mutually adjusted Se-78, Cu-63 and Zn-66 model for LDL-C; Table S21: LDL-C models excluding participants reporting current cholesterol medication; Table S22: Ni-60–homocysteine association after inverse-probability weighting for assay availability; Table S23: Model-standardized geometric means at illustrative serum trace-element contrasts; Table S24: Additional revision-stage multiplicity assessment across the 38 co-primary Model C comparisons; Figure S1: Cross-outcome effect heatmap; Figure S2: Serum element correlation heatmap; Figure S3: Hg-202 and LDL-C spline; Figure S4: Se-78 and LDL-C spline; Figure S5: Cu-63 and LDL-C spline; Figure S6: Zn-66 and LDL-C spline; Figure S7: Co-59 and NLR spline; Figure S8: Participant flow diagram; Figure S9: Alternative causal orderings relevant to interpretation of the cross-sectional associations; Combined STROBE and STROBE-ME reporting checklist.
