# Supplementary material for: Associations of Serum Trace-Element Concentrations with Selected Blood Biomarkers: A Secondary Cross-Sectional Analysis of the SPES Cohort
Source: Toxics. 2026 Sep 17;14(9):824. doi: 10.3390/toxics14090824 (PMC13611600; doi:10.3390/toxics14090824)
Supplement: Supplementary file 1 [file toxics-14-00824-s001.zip › toxics-4519207-supplementary.pdf]

Supplementary Materials

Associations of Serum Trace-Element Concentrations with Selected Blood Biomarkers: A Secondary Cross-Sectional Analysis of the SPES Cohort

Detailed methods, complete results, sensitivity analyses and supporting figures

Carlo Buonerba[1,*], Paolo Montuori[1], Ernesto Esposito[2], Maria Triassi[1], Pellegrino Cerino[1]

[1] Department of Public Health, University of Naples Federico II, Via Sergio Pansini 5, 80131 Naples, Italy; carbuone@hotmail.com (C.B.); pmontuor@unina.it (P.M.); triassi@unina.it (M.T.); pellegrinocerino@gmail.com (P.C.)

[2] Department of Health and Healthcare Services, Directorate-General of the Calabria Region, 88100 Catanzaro, Italy; ernesto.esposit1962@gmail.com (E.E.)

[*] Correspondence: carbuone@hotmail.com

Supplementary Methods

SM1. Data sources and analytic dataset

This secondary cross-sectional analysis used the curated de-identified SPES analytical dataset (4,188 participants; 257 variables) and 30 completed datasets generated under the parent study's censoring-aware multiple-imputation workflow. The analytical extract linked questionnaire variables, routine laboratory tests and 20 serum analytical channels representing 19 elements. Serum concentrations were treated as time-specific biomonitoring measurements. Essential-element concentrations reflect nutrition and host physiology as well as external exposure; concentration–marker associations do not establish toxicity or cumulative lifetime dose. Downstream association modelling used no participant-identifying fields. Medication questions were linked from the controlled parent questionnaire only to produce aggregate summaries and a cohort-exclusion mask; identifiers were not exported.

SPES recruited community residents in 2016–2017 from municipal population registries. Municipalities were grouped into 21 environmental pressure clusters, with a 4:2:1 allocation of recruitment targets across high-, medium- and low-pressure areas [25]. Recruitment used randomly selected registry records, mailed invitations, call-centre follow-up and territorial recruitment units [25–27]. The protocol specified ages 20–49 years, both sexes, informed consent, no reported history of HCV, HBV or HIV infection, and at least 10 continuous years of municipal residence [25]. Protocol eligibility criteria and observed sample characteristics are reported separately. The present primary cohort had a median age of 35 years (IQR 27–43), a range of 19–55 and 76 participants (1.9%) outside 20–49. The data required to calculate an invitation-based participation rate and systematically compare participants with nonparticipants were not collected; these assessments could not be provided.

The outcome hierarchy, covariate sets and multiplicity framework were specified before association modelling. No directional element-specific hypotheses were specified; the element panel was evaluated using two-sided tests within this predefined framework. The study was not externally preregistered. Supplementary Table S2 distinguishes specification, execution and subsequent exploratory additions. LDL-C was the sole lipid outcome modelled in this analysis. Prespecified and post-primary sensitivity analyses both use nominal inference; specification timing does not confer independent validation. The final analysis specification, analytic code, session information and aggregate outputs are retained by the authors and may be made available for editorial or reproducibility review on reasonable request, subject to study governance.

SM2. Cohort masks

Four non-imputed fields from the self-administered questionnaire recorded self-reported histories of physician-diagnosed tumour, myocardial infarction, angina and stroke. The tumour question did not establish validated malignant-cancer status. Explicit no/zero responses were required to classify each history as negative. Blank or missing responses remained unknown; other unrecognized responses were not treated as negative. Among 4,188 parent analytical records, 109 participants reported at least one history and 53 had incomplete history information (51 questionnaire nonresponders and 2 partial responders). The 4,135 participants with all four histories known comprised 109 with a positive history and 4,026 with all histories negative. The latter formed the primary cohort (96.1% of 4,188; 97.4% of 4,135). All-known-history sensitivity analyses retained participants with known histories regardless of positive status, assessing the consequences of the restriction without validating absence of unrecognized disease. The strict sensitivity cohort comprised 3,120 participants from the primary cohort who additionally reported no physician-diagnosed hypertension, dyslipidaemia (high cholesterol or triglycerides) or diabetes. Negative responses were required for all seven disease-history items; missing or unknown responses were not treated as absence of disease.

Before biospecimen collection, SPES performed a structured examination of general appearance; eyes, ears, nose and throat; neck and thyroid; lungs; heart; lymph nodes; abdomen and back; extremities; nervous system and reflexes; skin; and an additional "other" domain. Missing or unexamined domains were not classified as normal. Examination findings are summarized in SR1. The examination was not an additional exclusion criterion. Together with the self-administered questionnaire, it provided clinical information at enrolment, but these

sources could not systematically exclude every acute infection, inflammatory or haematological disorder, recent operation or treatment-related influence on blood counts.

SM3. Serum trace-element measurements, censoring and quality-control states

Each element channel was accompanied by assay-performed, censoring, LOQ and QC-high fields. Quantified values were finite positive concentrations from performed assays that were not classified below LOQ or QC-high. Left-censored values contributed to the censoring-aware completion models. Records exceeding element-specific quality-control upper bounds (QC-high) were set to missing, excluded from imputation-model fitting and donor pools and completed. Values from unperformed assays were imputed only when the proportion of unperformed assays among questionnaire responders was ≤5%. Hg and Ni exceeded this threshold, so analyses were restricted to assay-performed participants; unperformed Zn assays were eligible for completion. Assay counts are reported in SR2 and Table S5.

Assay nonperformance denotes an unperformed measurement and is distinct from a performed assay with a concentration below the LOQ. The operational reasons for Hg/Ni assay nonperformance could not be reconstructed. Differences in assay availability by environmental pressure area and municipal cluster (Table S16) describe observed patterns and do not establish why assays were unperformed. Weighting for measured covariates cannot eliminate selection related to unrecorded laboratory batch, calendar time, specimen availability or other factors.

The published SPES field procedure specified an instruction to fast for at least eight hours and a staff check before venepuncture; questionnaire, clinical assessment and blood collection occurred at the same enrolment encounter [27]. The serum-processing and ICP-MS procedures have been described previously [26,27]. Briefly, they used refrigerated centrifugation, storage at −80 °C under continuous temperature monitoring, a 500 μL serum aliquot, thawing/mixing and 1:10 dilution before duplicate NexION 350 ICP-MS measurements. The mean of duplicates was entered in the dataset. Daily five-point calibration, Y-89/Rh-103/Lu-175 internal standards, chemical blanks, internal quality controls and certified reference materials were used; LOQs were estimated from 20 reagent blanks [27]. Table S5 reports the channel-specific LOQs used in this analysis, with their units. Numerical within-/between-run precision, analyte-specific recoveries and raw replicate records were unavailable. QC-high flags were post-analytical plausibility exclusions, not documented instrument or batch QC failures. These procedural descriptions do not provide participant-level records of exact fasting duration, within-visit sampling intervals, collection dates or season, which were not retained in the present analytical extract.

Element interpretive classes were defined before association modelling: toxicological priority (As, Cd, Cr, Sb, Tl, V), measurement-limited (Be, Hg, Ni, Pb), essential/homeostatic (Co, Cu, Fe, Mn, Mo, Se, Zn) and other (Li, Sr). These classes governed presentation and interpretation, not multiplicity families. These descriptive classes do not rank harmfulness; measured concentrations of essential elements were not interpreted as toxic exposures.

All element concentrations refer to serum. They cannot be converted into whole-blood lead, urinary cadmium or species-specific exposure measures. Chemical speciation was unavailable for arsenic, chromium, mercury, selenium and antimony; an isotope/channel label does not identify a chemical species. Comparisons with other matrices were limited to qualitative context and do not establish equivalent dose, cumulative burden or clinical thresholds.

SM4. Laboratory-outcome construction

The outcomes were measured laboratory variables or indices calculated from blood cell counts, not incident disease endpoints. LDL-C represented lipid metabolism, NLR and SII were nonspecific count-derived indices, and PLR, RDW, glucose and homocysteine provided complementary laboratory outcomes. Associations with these outcomes do not establish a change in cancer or cardiovascular risk.

Absolute neutrophils and lymphocytes were recorded in $10^3/\mu L$. Platelets were recorded per μL and divided by 1,000 before ratio derivation. Within each completed dataset: NLR = neutrophils/lymphocytes; SII = (platelets/1,000) × neutrophils/lymphocytes; and PLR = (platelets/1,000)/lymphocytes. LDL-C was directly measured by the study laboratory and analysed as reported, without recalculation. Fasting instructions and the

pre-collection staff check are described in SM3. Exact individual fasting duration and sampling-time records were unavailable for assessing their influence on LDL-C and, particularly, glucose. NLR, SII, PLR, LDL-C, glucose, homocysteine and RDW were log-transformed in the main analysis. Native-unit sensitivity analyses were performed for LDL-C, glucose and RDW.
The count-derived panel comprised NLR, SII and PLR, calculated from the available absolute counts. NLR and SII do not directly measure innate or adaptive immune function, and inclusion of platelet counts does not measure platelet activity. RDW reflects variation in red cell size. These laboratory outcomes are nonspecific and can vary with infection, inflammatory or haematological disease, physiological stress and treatment. The structured examination is described in SM2 and its findings in SR1. The examination and self-administered questionnaire could not systematically exclude every condition affecting leukocyte or platelet counts. The self-reported medication questions and their reporting periods are described below; unavailable follow-up responses were not interpreted as absence of treatment.

CRP/hs-CRP, erythrocyte sedimentation rate and fibrinogen were unavailable. Total leukocyte and monocyte counts were recorded in the parent database but were not included in the retained analytical extract or the specified outcome hierarchy. CRP-based scores, derived NLR, lymphocyte-to-monocyte ratio, systemic inflammation response index and aggregate index of systemic inflammation were therefore not analysed. Albumin, ferritin, protein fractions, eosinophils and basophils were not selected as principal laboratory outcomes because of broader nutritional, hepatic, iron-metabolic, allergic and infectious determinants and to maintain a parsimonious multiplicity-controlled hierarchy.

Current medication for high blood pressure, current cholesterol medication and insulin or other diabetes medication were ascertained through self-reported follow-up questions linked to the corresponding physician-diagnosis items. Code 2 denoted yes and code 1 denoted no; unavailable follow-up responses were not interpreted as non-use. In the primary cohort, all three follow-ups were complete within their respective diagnosis groups: 180 yes/264 no among 444 reporting hypertension, 66 yes/514 no among 580 reporting high cholesterol or triglycerides and 22 yes/10 no among 32 reporting diabetes. The structured data did not reliably identify all individual drug classes; cholesterol medication was therefore not labelled as statin therapy. Medication variables were not included as regression adjustment covariates. The sensitivity analysis excluding the 66 identified current cholesterol medication users is reported in Table S21. No separate antihypertensive-exclusion sensitivity analysis was performed. Systematic data on dietary-supplement use, dose and duration were unavailable (SM7).

SM5. Multiple imputation

The parent SPES workflow used 135 substantive predictor candidates, including 41 routine laboratory fields, followed by deterministic exclusions and target-specific stabilization. The auxiliary pool included routine biochemical measures and blood-count components relevant to the present outcomes. Review of the initial recorded target-specific predictor selections showed inclusion of LDL-C for the Hg, Se, Cu and Zn censoring models; it did not establish that LDL-C remained in every subsequent fitting. Predictor use was distinct from imputation targeting: alcohol, physical activity, deprivation, energy intake, creatinine, glucose and homocysteine were complete in the 4,026-person primary cohort and were not imputed. Age, sex, impact-area stratum and municipal cluster were included in every imputation model. Routine laboratory variables with low missingness were imputed using predictive mean matching. Element values below LOQ were completed using family-specific censoring-aware models: Gaussian Tobit on the log scale (Family A), Gaussian Tobit on prespecified Box-Cox scales (Family B) or interval-censored Gamma regression (Family C). Unperformed assays were imputed only when assay nonperformance was at most 5%, with Hg and Ni excluded. Details of the parent workflow are provided in the previous SPES report [25,34–44].

The inherited imputations were not constructed specifically for the current substantive models. Inclusion in a broad candidate pool or in an initial predictor selection did not guarantee compatibility with every transformed outcome or derived blood-count ratio, and incompatibility could attenuate or otherwise distort associations. The

fraction of missing information describes coefficient-level imputation uncertainty; it does not test model compatibility. Main, directly quantified and applicable observed-CBC estimates are therefore reported together, with each restriction defined by the data component it addresses. These within-dataset sensitivities are not independent replication and can introduce selection through their restrictions.

SM6. Exposure scaling

Concentrations were expressed in μg/L, except Fe and Zn, which were expressed in mg/L. For element e and imputation m, exposure was $E_{iem} = [\log(M_{iem}) - c_e]/s_e$, where c_e was an arbitrary centring constant and s_e was the median of the 30 imputation-specific IQRs of log concentration in the primary cohort. The models were fitted on this fixed log-IQR scale. For presentation on a common relative concentration scale, $\beta_{doubling} = \beta_{log\text{-}IQR} \times \log(2)/s_e$; standard errors and coefficient-scale confidence limits were multiplied by the same factor. Percentage differences and their limits were then obtained as $100 \times [\exp(\beta_{doubling}) - 1]$. The p and q values were unchanged. A doubling is a common relative increment, not an equal absolute dose or an index of comparative biological potency. The original log-IQR estimates and approximate concentration contrasts remain in Tables S6–S10 and S19. For channels with at least 25% left-censoring, empirical Q1 was below the LOQ and is displayed as <LOQ rather than as a measured numerical quartile. Quantification-status comparisons and spline analyses retain their own exposure definitions.

Model-standardized geometric means were calculated to describe the magnitude of selected fitted associations in the outcome's original units. Within each completed dataset, each stated element concentration was assigned to all participants while their other covariates were retained. The fully adjusted Model C linear predictors were averaged over the relevant analysis cohort and exponentiated; the resulting geometric means were pooled across the 30 completed datasets. For LDL-C, these summaries are expressed in mg/dL and are not arithmetic means. Panel A of Table S23 uses one fixed log-IQR anchored at reconstructed Q1; Panel B uses the below-LOQ versus quantifiable contrast among assay-performed participants. The same conditional interpretation applies to SII, RDW and homocysteine in their own units. These are cohort-level summaries of conditional associations, not estimates of individual effects, causal intervention effects, biological cut-offs or treatment targets. Standardization does not resolve temporality or residual confounding. The selected concentrations are illustrative contrasts; the summaries do not add independent hypothesis tests or alter the fitted coefficients or their original multiplicity adjustments.

SM7. Model specification and clustered inference

Model A included exposure, age modelled with a restricted cubic spline using knots at the 10th, 50th and 90th percentiles, sex and 21-level municipal-cluster fixed effects. Model B additionally included BMI modelled with a restricted cubic spline, smoking status, log1p alcohol, log1p physical activity, deprivation score, occupational exposure and log1p energy intake. Model C added element-specific source variables: Hg, fish and shellfish; As, fish, shellfish and rice; Cd and Pb, dietary iron and calcium; Fe, dietary iron; Zn, dietary zinc. All dietary-source quantities were entered as log(1 + intake). For all other elements, Model C used the common Model B covariates. The Italian EPIC food-frequency questionnaire referred to habitual intake over the preceding year. Fish and shellfish quantities were separate g/day covariates, each transformed as log(1 + intake). Total energy intake and the socioeconomic covariates remained in the common adjustment set. The retained dietary data did not distinguish species, provenance, preparation or recent seafood intake, and separate validation coefficients for the aggregated fish and shellfish variables were unavailable. This adjustment could not eliminate residual confounding by seafood-associated nutrients or dietary patterns.

Validated selenium and copper intake estimates and comprehensive supplement use, dose and duration data were unavailable. Residual nutritional confounding, particularly for Se, Cu and Zn, could therefore remain despite the available food and energy covariates.

Models were ordinary least squares regressions on the transformed outcome. HC1 sandwich covariance estimates were clustered by municipality. A model required at least 200 participants and 30 municipalities. Complete-data degrees of freedom were G−1, where G was the number of municipalities in the common frame.

Coefficients and within-imputation robust variances were pooled using Rubin and Barnard–Rubin rules. The exponentiated coefficient was reported as 100 × [exp(β) − 1].

Explanatory causal assumptions. The fitted coefficients describe covariate-adjusted cross-sectional associations, not identified total or direct causal effects. Supplementary Figure S9 illustrates alternative temporal assumptions: Panel A includes a hypothesized serum-concentration-to-outcome pathway and shared causes, whereas Panel B includes the reverse pathway, physiological intermediates of earlier exposure and selection under the disease-history restriction. In Panel A, diet and supplements, BMI, renal physiology and inflammatory physiology are potential common causes of serum concentrations and laboratory outcomes. In Panel B, physiological factors may mediate effects of earlier external exposure; this does not establish mediation of an effect of the contemporaneously measured serum concentration. These broad conceptual groups are not a fully observed adjustment set; creatinine entered only the renal sensitivity analysis. The existing no-BMI and creatinine-adjusted sensitivities assess changes in model specification but cannot determine which causal ordering is correct. The diagrams were constructed during revision to make these interpretive assumptions explicit; they did not determine the original adjustment set.

SM8. Multiplicity and nonlinear analyses

The predefined outcome hierarchy organized discovery-oriented, two-sided testing of the element panel; it did not encode directional hypotheses for particular elements. Timing of specification and execution is summarized in Supplementary Table S2. Benjamini–Hochberg correction was applied to NLR and LDL-C separately (19 element tests each), SII (19), PLR/RDW jointly (38), glucose (19) and exploratory homocysteine (19). The co-primary outcome-specific families supported endpoint-specific inference; no either-endpoint combined success criterion or cross-domain claim was applied. Fe-57 consistency analyses were outside these families. Restricted cubic spline analyses were secondary. Four separate 17-test BH families covered LDL-C global association, LDL-C nonlinearity, NLR global association and NLR nonlinearity, after excluding Be and Pb because their continuous lower tails were predominantly model-generated. Secondary and exploratory analyses retained those designations despite family-specific multiplicity control. Sensitivity p values were nominal and were interpreted through estimate magnitude, direction and uncertainty, rather than as independent confirmation. The revision-stage 38-test correction applied only to the combined co-primary linear family.

A revision-stage assessment applied Benjamini–Hochberg correction to the 38 original unrounded Model C p values across LDL-C and NLR together. The original 19-test outcome-specific q values were retained. This calculation did not refit any model or change coefficients, confidence intervals or original p values. The combined primary correction did not extend to secondary or sensitivity analyses. Supplementary Table S24 reports outcome, serum element, original Model C p value, original within-outcome q value, combined 38-test q value and whether $q < 0.05$ under each definition.

The stated FDR corrections apply only to their specified hypothesis families. No additional multiplicity correction was applied to sensitivity analyses, including the three post-primary exploratory additions. Their findings were evaluated by estimate magnitude, direction and uncertainty rather than treated as additional validated positive results. Per-doubling results and model-standardized summaries re-express existing fitted results and do not constitute independent validation.

SM9. Sensitivity analysis programme

The original sensitivity programme was specified before association modelling. Its estimates and confidence intervals were interpreted using nominal inference to assess magnitude, direction and uncertainty, not as independent validation or additional multiplicity-controlled discoveries. CR2 covariance and influence diagnostics followed predefined triggering rules and are distinguished from routinely executed sensitivities. The three post-primary exploratory additions also used nominal inference and are identified below. The supplementary chronology separates specification, execution and descriptive re-expression of fitted results; the study was not externally preregistered.

Supplementary Table S1. Sensitivity analysis definitions.

Analysis	Purpose/implementation
Observed CBC	NLR, SII and PLR were reconstructed only from original observed constituent cell counts; RDW used original observed RDW values. This did not require serum element concentrations to be directly quantified.
Quantification status	Performed assays classified as quantifiable were compared with those below LOQ using observed status, without assigning numerical below-LOQ concentrations for that predictor.
Direct quantified	Models restricted the element predictor to original, directly quantified concentrations. LDL-C was observed in all 4,026 primary-cohort participants; directly quantified LDL-C models therefore used observed element concentrations and observed LDL-C. The reporting unit remained the fixed original element-specific log-IQR.
Alternative populations	All known history, strict sensitivity, known negative tumour history and known negative MI/angina/stroke history masks were evaluated (SM2).
Covariate structure	Models omitted BMI or deprivation, added creatinine, excluded occupationally exposed participants, or replaced cluster with municipality fixed effects.
Outcome-treatment proxies	LDL-C was reanalysed after excluding reported dyslipidaemia; glucose after excluding diabetes.
Exposure-specific restrictions	Current smokers were excluded for Cd/Pb; QC-high participants were excluded by channel.
Native units	LDL-C, glucose, and RDW were analysed on their native scales.
Covariance	CR2 covariance with small-sample correction was applied to FDR-significant primary associations.
Assay availability	Hg and Ni assay availability was described by standardized differences and examined using inverse-probability-of-observation weighting. Separate logistic models for assay availability were fitted in the 4,188-record parent analytical dataset with age (restricted cubic spline), sex, geographical cluster, questionnaire-response status, smoking status (including an unknown category), BMI (with median replacement of missing values), deprivation and corresponding missingness indicators, creatinine, LDL-C, glucose and homocysteine. Both models used cluster because the criterion for including municipality—both assay availability states in every municipality—was not met. Predicted probabilities were bounded at 0.01 and 0.99. Among participants with the assay, stabilized weights equalled the observed assay availability proportion divided by the predicted probability; weights were then truncated at their 1st and 99th percentiles before application to the relevant analytical cohort. This sensitivity analysis addresses selection explained by the observed covariates in the weighting models; unmeasured determinants of assay availability may remain.
Alternative Family C	Li, Co, Cu, Fe-57, Mo, and Sb were reanalysed using the alternative Family-C completion set.
Influence	Prespecified trimming and influence diagnostics assessed whether a small number of observations drove FDR-significant primary results.

Three post-primary exploratory sensitivity analyses evaluated (i) LDL-C after excluding 66 participants identified by the questionnaire as current cholesterol medication users, (ii) the joint Se-78/Cu-63/Zn-66–LDL-C model and (iii) Ni-60–homocysteine after assay availability weighting, extending the original Hg/Ni weighting programme to this exploratory outcome. All used nominal inference and did not alter the primary FDR families. The original programme retained the alternative Family-C completion analysis and the other analyses listed in Table S1. Influence sensitivity was defined as direction reversal or a relative change in effect magnitude greater than 25%.

A formal whole-panel mixture analysis was not specified. The joint Se/Cu/Zn–LDL-C model was a post-primary exploratory sensitivity analysis of a correlated essential-element block. Its coefficients describe conditional associations, and neither the individual coefficients nor the global block test identifies independent causal effects or an overall pollutant-mixture effect. The three exposures retained their original fixed log-IQR scales. The joint model included the common Model B covariates and dietary zinc (Table S20); individual Se and Cu models did

not include dietary zinc, so differences between their estimates and the joint estimates cannot be attributed solely to mutual adjustment for serum elements.

Supplementary Table S2. Timing and inferential status of the analysis programme.

Analysis or decision	Timing	Purpose and status
Outcome hierarchy, adjustment and multiplicity framework; LDL-C as the sole lipid outcome	Before association modelling; LDL-C-only amendment on 28 July 2026	Primary analytical framework; LDL-C was the only lipid outcome modelled; no external preregistration
Original sensitivity programme	Specified before association modelling	Robustness assessments defined before primary results
Cluster-robust CR2 and influence diagnostics	Triggering rules specified before modelling; execution conditional on diagnostics	Conditional diagnostics within the original programme
Joint Se/Cu/Zn–LDL-C model; cholesterol medication exclusion; Ni–homocysteine assay availability weighting (extension of the original Hg/Ni weighting programme to this exploratory outcome)	29 July 2026, after the primary analysis	Additional exploratory analyses; nominal inference
Per-doubling translations; model-standardized geometric means	29 July and 3 August 2026, respectively	Re-expression and interpretation of fitted results; no new hypothesis tests
Combined BH adjustment across 38 co-primary comparisons	During revision	Additional multiplicity assessment of existing primary p values; no new model fits

SM10. Software and reproducibility

Association analyses were conducted in R 4.6.1 on Windows 11. The primary-analysis random-number seed was 20260728; additional exploratory sensitivities used 20260729. Key package versions were mice 3.19.0, sandwich 3.1-2, clubSandwich 0.7.0, ggplot2 4.0.3, dplyr 1.2.1, readxl 1.5.0, survival 3.8-6, MASS 7.3-65, Matrix 1.7-5 and yaml 2.3.12. Descriptive calculation of model-standardized geometric means was performed in R 4.5.1 using the same frozen imputation object and Model C specification. The complete analysis specification, code, session information and aggregate outputs are retained by the authors and may be made available for editorial or reproducibility review on reasonable request, subject to study governance.

Supplementary Results

SR1. Cohort reconciliation and clinical characteristics

The parent programme enrolled 4,205 participants; 17 outside the designated area were excluded, yielding 4,188 analytical records, including 4,137 questionnaire responders and 51 nonresponders. All four specified histories were known for 4,135 participants. Of these, 109 reported at least one physician-diagnosed tumour, myocardial infarction, angina or stroke, and 4,026 reported none. Another 53 had incomplete history information. Three primary-cohort participants lacked BMI, leaving 4,023 in most Model C analyses. Hg scaling used 3,259 assay-performed participants, with 3,258 in Model C because one lacked BMI; Ni used 2,213 for both scaling and modelling. An examination was recorded as performed for all 4,026 participants. General appearance was normal in 3,980 of 4,025 assessed participants (98.9%). Among 4,013 participants with all 11 examination domains assessed, 3,932 (98.0%) had no clinically significant abnormality in any domain.

For the diagnosis-linked follow-up questions, the identified users numbered 180 for current high-blood-pressure medication, 66 for current cholesterol medication and 22 for insulin or another diabetes medication, among diagnosis groups of 444, 580 and 32 participants, respectively. Missing follow-up responses were not interpreted as non-use. The counts do not establish complete ascertainment of treatment at blood collection. The questionnaire counts are reported in Table S18.

SR2. Assay support and completion precision

Assay availability was high for most channels but substantially lower for Hg and Ni. In the full 4,188-participant analytical cohort, 785 Hg assays (18.7%), 1,861 Ni assays (44.4%) and 180 Zn assays (4.3%) were unperformed (Table S5). Hg/Ni assay availability differed by environmental pressure area and municipal cluster (Table S16); these patterns do not establish the operational reasons for nonperformance. Left-censoring exceeded 40% for Be, Pb, Cd and Cr. Coefficient-level imputation uncertainty was modest: the maximum fraction of missing information was 0.142 within the two primary outcome families and 0.177 across all Model C analyses.

SR3. Complete primary and secondary results

The complete 19-element Model C estimates are reported in Supplementary Tables S6–S10 on both the population log-IQR scale and a common two-fold concentration scale. Four LDL-C associations, four SII associations, one RDW association and two exploratory homocysteine associations passed FDR correction. PLR and RDW comprised a single 38-test family; the Fe-56 RDW q value reflects correction across both outcomes.

SR4. Nonlinearity and correlation structure

The LDL-C global spline tests were significant for Hg, Se, Cu and Zn, but no test for nonlinearity survived FDR correction. No primary linear element–NLR association met the original outcome-specific FDR threshold or the additional BH threshold across the 38 co-primary comparisons. The secondary global Co spline test was FDR-significant, whereas its specifically nonlinear component was not significant. These are different tests and do not establish the absence of association. The inter-element correlation structure is shown in Figure S2, and the largest pairwise correlations are listed in Table S15.

SR5. Sensitivity analyses

All four FDR-significant LDL-C associations remained directionally consistent across cohort, covariate, covariance, geographic and medication-exclusion analyses. Excluding 66 identified current cholesterol medication users yielded +3.49% for Hg, +2.18% for Se, +1.65% for Cu and +1.52% for Zn per original log-IQR. For Hg, the observed quantifiable-versus-below-LOQ contrast was +4.08% (95% CI 1.77 to 6.43), whereas the continuous estimate within directly quantified concentrations was +1.89% per original fixed log-IQR (−1.46 to 5.36; $p = 0.269$). The Hg quantification-status comparison was positive, while the imprecise continuous estimate among directly quantified concentrations did not demonstrate a clear gradient. The two analyses use different exposure contrasts and sample restrictions; a positive status comparison does not validate a continuous dose–response relationship. In the post-primary joint Se/Cu/Zn model ($n = 4{,}023$), Se had a positive conditional association with LDL-C (+1.56% per original log-IQR; 95% CI 0.47 to 2.66), while Cu and Zn estimates were smaller and their confidence intervals included zero (Table S20). These nominal exploratory estimates do not identify independent causal effects; the differing dietary zinc adjustment also prevents attributing the differences from individual Se/Cu estimates solely to mutual adjustment. The post-primary assay availability weighting analysis for Ni-60 and homocysteine produced +3.34% per original fixed log-IQR (1.44 to 5.28; $p = 6.12e{-}04$), with an effective sample size of 1,991. Assay availability weighting produced estimates close to the primary results: Hg–LDL-C changed from +3.72% to +3.63%, and Ni–homocysteine from +3.36% to +3.34%, per original element-specific log-IQR (Tables S13 and S22). These comparisons indicate little sensitivity to reweighting using the measured covariates in the assay availability models, while selection related to unmeasured factors remains possible. SII sensitivity results were heterogeneous: the continuous Sb and Co estimates among directly quantified concentrations were imprecise; Cu attenuated in the strict sensitivity cohort, and Mo remained positive. Sensitivity estimates use nominal inference and do not provide independent validation.

SR6. Model-standardized serum concentration–marker associations

Table S23 reports cohort-standardized geometric means at illustrative serum element contrasts. For continuous channels, the contrast is one fixed log-IQR anchored at reconstructed Q1; for Hg–LDL-C, Sb–SII and Ni–homocysteine, the table also reports below-LOQ versus quantifiable contrasts among assay-performed participants. The values describe the magnitude of conditional associations in original outcome units. They do not estimate individual changes, causal intervention effects, biological cut-offs or treatment targets.

SR7. Selected external human-evidence context

External human evidence was used only to contextualize the findings and was not used to select outcomes or models. The main Discussion section summarizes supportive and contrasting prospective, cross-sectional and intervention evidence while distinguishing differences in population, biological matrix, exposure scale and endpoint. Randomized supplementation evidence was used to constrain intervention claims, not to treat serum concentration variation as equivalent to supplementation. The present findings do not justify selenium, copper or zinc restriction or supplementation for lipid management. Prospective repeated-measures studies, improved dietary and treatment assessment, defined mixture questions and appropriately validated genetic instruments are future research priorities; none is presented as an analysis performed in this report.

Supplementary Tables

Supplementary Table S3. Cohort counts.

Population/mask	n
Parent programme enrolled	4205
Outside designated area	17
Parent analytical dataset	4188
Incomplete information on ≥1 of the four histories	53
Primary cohort	4026
All four disease-history fields known	4135
Any self-reported physician-diagnosed tumour/MI/angina/stroke	109
Strict sensitivity cohort	3120
Known negative physician-diagnosed tumour history	4051
Known negative MI/angina/stroke history	4112
Questionnaire responders	4137
Questionnaire nonresponders	51

The primary cohort represented 96.1% of the 4,188 parent analytical records and 97.4% of the 4,135 with all four histories known. The strict sensitivity cohort comprised 3,120 participants from the primary cohort who additionally reported no physician-diagnosed hypertension, dyslipidaemia (high cholesterol or triglycerides) or diabetes. Negative responses were required for all seven disease-history items; missing or unknown responses were not treated as absence of disease. These labels describe self-report, not clinically verified disease-free status.

Supplementary Table S4. Primary cohort characteristics.

Variable	Level/statistic	Value	Observed/known n	Missing/unknown n	Imputation status
Age	Continuous	35.00 [27.00-43.00]	4026	0	No
Body mass index	Continuous	24.62 [22.23-27.68]	4023	3	No; residual model exclusion
Alcohol intake	Continuous	1.25 [0.15-7.11]	4026	0	No
Physical activity	Continuous	36.00 [15.75-66.75]	4026	0	No
Socioeconomic deprivation score	Continuous	29.20 [8.30-37.50]	4026	0	No
Total energy intake	Continuous	1890.03 [1461.52-2422.03]	4026	0	No
Creatinine	Continuous	0.80 [0.70-0.90]	4026	0	No
LDL-C	Continuous	116.00 [95.00-140.00]	4026	0	No

Variable	Level/statistic	Value	Observed/known n	Missing/unknown n	Imputation status
Glucose	Continuous	77.00 [72.00-83.00]	4026	0	No
Homocysteine	Continuous	8.10 [7.20-9.35]	4026	0	No
Observed NLR	Continuous	1.60 [1.24-2.05]	3943	83	Derived after constituent-count imputation
Observed SII	Continuous	363.87 [269.83-493.49]	3941	85	Derived after constituent-count imputation
Observed PLR	Continuous	104.40 [83.94-130.06]	3941	85	Derived after constituent-count imputation
Observed RDW	Continuous	12.04 [11.61-12.60]	3943	83	Predictive mean matching
Sex	Female	2165 (53.8%)	4026	0	No
Sex	Male	1861 (46.2%)	4026	0	No
Smoking status	Never	2050 (50.9%)	4026	0	No
Smoking status	Former	730 (18.1%)	4026	0	No
Smoking status	Current	1246 (30.9%)	4026	0	No
Occupational exposure	No	3224 (80.1%)	4026	0	No
Occupational exposure	Yes	802 (19.9%)	4026	0	No
Municipal cluster	1	198	4026	0	No
Municipal cluster	2	192	4026	0	No
Municipal cluster	3	197	4026	0	No
Municipal cluster	4	196	4026	0	No
Municipal cluster	5	195	4026	0	No
Municipal cluster	6	195	4026	0	No
Municipal cluster	7	191	4026	0	No
Municipal cluster	8	193	4026	0	No
Municipal cluster	9	199	4026	0	No
Municipal cluster	10	196	4026	0	No
Municipal cluster	11	192	4026	0	No
Municipal cluster	12	196	4026	0	No
Municipal cluster	13	173	4026	0	No
Municipal cluster	14	204	4026	0	No
Municipal cluster	15	202	4026	0	No
Municipal cluster	16	193	4026	0	No
Municipal cluster	17	197	4026	0	No
Municipal cluster	18	187	4026	0	No
Municipal cluster	19	176	4026	0	No
Municipal cluster	20	166	4026	0	No
Municipal cluster	21	188	4026	0	No

Continuous values are median [IQR]. Percentages for categorical variables use the primary-cohort denominator unless the item is mask-specific. Observed/known and missing/unknown counts refer to original primary-cohort records (N = 4,026); BMI counts refer to availability for the primary models after the original data preparation. For NLR, SII and PLR, availability requires every constituent count used in that index. Descriptive laboratory summaries use observed measurements. RDW and the constituent blood counts were eligible for predictive-mean-matching imputation; the indices were derived after component completion. Completed-data model n can differ from observed-data n because of imputation, exposure availability and residual covariate missingness. Medication follow-up

denominators comprise participants reporting the corresponding diagnosis; blanks outside that stratum do not establish non-use. Element-assay support in Table S5 uses its separately labelled full-cohort denominator of 4,188 and distinguishes unperformed assays, below-LOQ measurements and post-analytical plausibility exclusions.

Supplementary Table S5. Element measurement support.

Panel A. Measurement support.

Channel	Measured, n	Unperformed, %	<LOQ among measured, %	Quantified, n	QC-high, n	Class
Li-7	4188	0.0%	0.6%	4161	3	Other
Be-9	4188	0.0%	69.2%	1289	0	Measurement-limited
Cd-111	4188	0.0%	44.7%	2318	0	Toxicological priority
As-75	4187	0.0%	7.0%	3892	0	Toxicological priority
Cr-52	4185	0.1%	41.1%	2467	0	Toxicological priority
Co-59	4185	0.1%	1.0%	4143	0	Essential/homeostatic
Cu-63	4185	0.1%	0.0%	4184	0	Essential/homeostatic
Fe-56	4185	0.1%	0.2%	4175	0	Essential/homeostatic
Hg-202	3403	18.7%	25.5%	2530	6	Measurement-limited
Mn-55	4187	0.0%	10.4%	3753	0	Essential/homeostatic
Mo-98	4188	0.0%	15.2%	3547	3	Essential/homeostatic
Ni-60	2327	44.4%	30.5%	1612	6	Measurement-limited
Pb-208	4188	0.0%	71.0%	1211	4	Measurement-limited
Sb-121	4188	0.0%	36.2%	2665	5	Toxicological priority
Se-78	4188	0.0%	0.0%	4187	0	Essential/homeostatic
Sr-88	4188	0.0%	0.0%	4187	0	Other
Tl-205	4188	0.0%	25.7%	3110	1	Toxicological priority
V-51	4185	0.1%	11.7%	3688	9	Toxicological priority
Zn-66	4008	4.3%	0.0%	4007	0	Essential/homeostatic
Fe-57	4185	0.1%	0.2%	4176	0	Analytical consistency

Panel B. Channel-specific limits of quantification.

Channel	LOQ	Unit
Li-7	0.0048	μg/L
Be-9	0.0015	μg/L
Cd-111	0.0010	μg/L
As-75	0.0015	μg/L
Cr-52	0.0015	μg/L
Co-59	0.0016	μg/L
Cu-63	0.3007	μg/L
Fe-56	0.0007	mg/L
Hg-202	0.1192	μg/L
Mn-55	0.0096	μg/L
Mo-98	0.0099	μg/L
Ni-60	0.0212	μg/L

Channel	LOQ	Unit
Pb-208	0.0182	μg/L
Sb-121	0.0020	μg/L
Se-78	0.1114	μg/L
Sr-88	0.0193	μg/L
Tl-205	0.0012	μg/L
V-51	0.0087	μg/L
Zn-66	0.0004	mg/L
Fe-57	0.0006	mg/L

LOQs are analytical quantification limits, not clinical thresholds. Fe and Zn values use mg/L; other channels use μg/L. Assay-nonperformance percentages use all 4,188 participants in the parent analytical cohort; left-censoring percentages use performed assays. Unperformed assays are distinct from performed assays below LOQ. Hg/Ni analyses were restricted to assay-performed participants; Tables S6–S10 report outcome-specific model denominators. QC-high indicates a post-analytical plausibility screen rather than a recorded assay-batch failure. Fe-57 is a duplicate iron channel outside the 19-element primary multiplicity families.

Supplementary Table S6. Complete Model C results for NLR.

Serum element	n	Per log-IQR, % (95% CI)	Per doubling, % (95% CI)	p	q	Class
Li-7	4023	-0.60% (-1.76% to +0.57%)	-0.59% (-1.74% to +0.56%)	0.311	0.493	Other
Be-9	4023	-0.71% (-2.33% to +0.94%)	-0.20% (-0.67% to +0.27%)	0.394	0.576	Measurement-limited
Cd-111	4023	+0.76% (-1.69% to +3.27%)	+0.11% (-0.25% to +0.48%)	0.543	0.645	Toxicological priority
As-75	4023	-0.70% (-1.68% to +0.29%)	-0.28% (-0.69% to +0.12%)	0.166	0.351	Toxicological priority
Cr-52	4023	+3.13% (+0.63% to +5.70%)	+0.31% (+0.06% to +0.56%)	0.014	0.109	Toxicological priority
Co-59	4023	+0.95% (+0.25% to +1.65%)	+1.23% (+0.33% to +2.15%)	0.008	0.109	Essential/homeostatic
Cu-63	4023	+0.34% (-0.74% to +1.43%)	+0.72% (-1.58% to +3.08%)	0.539	0.645	Essential/homeostatic
Fe-56	4023	-0.38% (-2.88% to +2.18%)	-0.47% (-3.55% to +2.71%)	0.768	0.811	Essential/homeostatic
Hg-202	3258	+0.22% (-3.20% to +3.76%)	+0.05% (-0.73% to +0.84%)	0.901	0.901	Measurement-limited
Mn-55	4023	+0.78% (+0.06% to +1.51%)	+0.56% (+0.04% to +1.08%)	0.035	0.132	Essential/homeostatic
Mo-98	4023	+0.84% (+0.02% to +1.67%)	+0.45% (+0.01% to +0.89%)	0.045	0.144	Essential/homeostatic
Ni-60	2213	+3.74% (+0.59% to +6.98%)	+0.52% (+0.08% to +0.96%)	0.020	0.109	Measurement-limited
Pb-208	4023	+0.49% (-0.85% to +1.85%)	+0.21% (-0.37% to +0.79%)	0.474	0.643	Measurement-limited
Sb-121	4023	+3.24% (+0.45% to +6.10%)	+0.45% (+0.06% to +0.84%)	0.023	0.109	Toxicological priority
Se-78	4023	-0.74% (-2.08% to +0.61%)	-1.41% (-3.92% to +1.17%)	0.280	0.493	Essential/homeostatic
Sr-88	4023	-1.01% (-2.33% to +0.33%)	-1.55% (-3.56% to +0.51%)	0.138	0.329	Other
Tl-205	4023	+0.64% (-2.08% to +3.44%)	+0.12% (-0.40% to +0.64%)	0.647	0.723	Toxicological priority
V-51	4023	+0.56% (-0.49% to +1.62%)	+0.33% (-0.29% to +0.94%)	0.298	0.493	Toxicological priority
Zn-66	4023	-0.94% (-2.02% to +0.16%)	-2.04% (-4.37% to +0.35%)	0.093	0.253	Essential/homeostatic

The NLR family comprised 19 element tests. Log-IQR estimates represent element-specific distribution contrasts. Per-doubling estimates use a common relative concentration increment; neither scale establishes comparative biological potency. Algebraic rescaling leaves p and q values unchanged. Approximate nominal two-sided 80% minimum detectable differences ranged from 0.98% to 5.05% per original log-IQR. These calculations used $\alpha = 0.05$ and did not incorporate the data-dependent BH rejection threshold. They describe approximate nominal precision, not observed power or effects excluded after multiplicity correction.

Supplementary Table S7. Complete Model C results for LDL-C.

Serum element	n	Per log-IQR, % (95% CI)	Per doubling, % (95% CI)	p	q	Class
Li-7	4023	-0.55% (-1.31% to +0.21%)	-0.54% (-1.30% to +0.21%)	0.157	0.331	Other
Be-9	4023	-0.58% (-1.80% to +0.66%)	-0.17% (-0.52% to +0.19%)	0.353	0.412	Measurement-limited
Cd-111	4023	-0.06% (-1.65% to +1.56%)	-0.01% (-0.25% to +0.23%)	0.944	0.944	Toxicological priority
As-75	4023	+0.82% (+0.15% to +1.50%)	+0.33% (+0.06% to +0.61%)	0.017	0.065	Toxicological priority
Cr-52	4023	-0.71% (-2.24% to +0.85%)	-0.07% (-0.23% to +0.09%)	0.369	0.412	Toxicological priority
Co-59	4023	-0.17% (-0.68% to +0.35%)	-0.22% (-0.88% to +0.45%)	0.519	0.548	Essential/homeostatic
Cu-63	4023	+1.55% (+0.74% to +2.36%)	+3.33% (+1.58% to +5.12%)	2.14e-04	0.002	Essential/homeostatic
Fe-56	4023	+0.62% (-0.46% to +1.71%)	+0.77% (-0.56% to +2.12%)	0.257	0.376	Essential/homeostatic
Hg-202	3258	+3.72% (+1.69% to +5.79%)	+0.83% (+0.38% to +1.28%)	3.47e-04	0.002	Measurement-limited
Mn-55	4023	-0.29% (-0.86% to +0.29%)	-0.20% (-0.62% to +0.21%)	0.328	0.412	Essential/homeostatic
Mo-98	4023	-0.55% (-1.38% to +0.28%)	-0.30% (-0.74% to +0.15%)	0.191	0.363	Essential/homeostatic
Ni-60	2213	-2.21% (-4.78% to +0.42%)	-0.32% (-0.69% to +0.06%)	0.099	0.268	Measurement-limited
Pb-208	4023	-0.87% (-1.71% to -0.02%)	-0.37% (-0.73% to -0.01%)	0.044	0.139	Measurement-limited
Sb-121	4023	+1.31% (-0.42% to +3.07%)	+0.18% (-0.06% to +0.43%)	0.137	0.326	Toxicological priority
Se-78	4023	+2.04% (+1.14% to +2.95%)	+3.93% (+2.18% to +5.70%)	1.30e-05	2.47e-04	Essential/homeostatic
Sr-88	4023	-0.59% (-1.76% to +0.60%)	-0.90% (-2.69% to +0.92%)	0.327	0.412	Other
Tl-205	4023	+1.15% (-0.81% to +3.15%)	+0.22% (-0.15% to +0.59%)	0.252	0.376	Toxicological priority
V-51	4023	-0.49% (-1.29% to +0.31%)	-0.29% (-0.76% to +0.18%)	0.229	0.376	Toxicological priority
Zn-66	4023	+1.26% (+0.49% to +2.04%)	+2.78% (+1.08% to +4.51%)	0.001	0.007	Essential/homeostatic

The LDL-C family comprised 19 element tests. Log-IQR estimates represent element-specific distribution contrasts. Per-doubling estimates use a common relative concentration increment; neither scale establishes comparative biological potency. Algebraic rescaling leaves p and q values unchanged.

Supplementary Table S8. Complete Model C results for SII.

Serum element	n	Per log-IQR, % (95% CI)	Per doubling, % (95% CI)	p	q	Class
Li-7	4023	-1.09% (-2.43% to +0.28%)	-1.07% (-2.40% to +0.28%)	0.118	0.306	Other
Be-9	4023	-0.59% (-2.32% to +1.17%)	-0.17% (-0.67% to +0.33%)	0.507	0.642	Measurement-limited
Cd-111	4023	+1.42% (-1.63% to +4.55%)	+0.21% (-0.24% to +0.66%)	0.363	0.596	Toxicological priority
As-75	4023	-0.53% (-1.71% to +0.66%)	-0.22% (-0.70% to +0.27%)	0.376	0.596	Toxicological priority
Cr-52	4023	+2.31% (-0.64% to +5.35%)	+0.23% (-0.06% to +0.53%)	0.125	0.306	Toxicological priority
Co-59	4023	+1.46% (+0.65% to +2.28%)	+1.90% (+0.84% to +2.97%)	4.89e-04	0.009	Essential/homeostatic
Cu-63	4023	+2.34% (+0.64% to +4.07%)	+5.07% (+1.37% to +8.89%)	0.007	0.034	Essential/homeostatic
Fe-56	4023	-0.53% (-3.36% to +2.37%)	-0.66% (-4.14% to +2.94%)	0.714	0.754	Essential/homeostatic
Hg-202	3258	+1.42% (-2.72% to +5.74%)	+0.32% (-0.62% to +1.27%)	0.504	0.642	Measurement-limited
Mn-55	4023	+0.70% (-0.20% to +1.61%)	+0.50% (-0.15% to +1.15%)	0.129	0.306	Essential/homeostatic
Mo-98	4023	+1.39% (+0.44% to +2.35%)	+0.74% (+0.24% to +1.25%)	0.004	0.032	Essential/homeostatic
Ni-60	2213	+4.69% (+0.87% to +8.65%)	+0.65% (+0.12% to +1.18%)	0.016	0.061	Measurement-limited
Pb-208	4023	+0.41% (-1.09% to +1.94%)	+0.18% (-0.47% to +0.82%)	0.591	0.702	Measurement-limited
Sb-121	4023	+4.29% (+1.29% to +7.38%)	+0.59% (+0.18% to +1.01%)	0.005	0.032	Toxicological priority
Se-78	4023	+1.15% (-0.45% to +2.78%)	+2.21% (-0.85% to +5.37%)	0.158	0.333	Essential/homeostatic
Sr-88	4023	-0.53% (-2.05% to +1.01%)	-0.82% (-3.13% to +1.55%)	0.494	0.642	Other

Serum element	n	Per log-IQR, % (95% CI)	Per doubling, % (95% CI)	p	q	Class
Tl-205	4023	+0.58% (-2.32% to +3.56%)	+0.11% (-0.44% to +0.67%)	0.697	0.754	Toxicological priority
V-51	4023	-0.20% (-1.50% to +1.11%)	-0.12% (-0.88% to +0.65%)	0.758	0.758	Toxicological priority
Zn-66	4023	-0.91% (-2.26% to +0.46%)	-1.98% (-4.87% to +1.00%)	0.189	0.360	Essential/homeostatic

The SII family comprised 19 element tests. Log-IQR estimates represent element-specific distribution contrasts. Per-doubling estimates use a common relative concentration increment; neither scale establishes comparative biological potency. Algebraic rescaling leaves p and q values unchanged.

Supplementary Table S9. Complete Model C results for PLR and RDW.

Laboratory outcome	Serum element	n	Per log-IQR, % (95% CI)	Per doubling, % (95% CI)	p	q	Class
PLR	Li-7	4023	-0.89% (-1.85% to +0.09%)	-0.87% (-1.83% to +0.09%)	0.075	0.286	Other
PLR	Be-9	4023	-0.03% (-1.33% to +1.28%)	-0.01% (-0.38% to +0.36%)	0.965	0.965	Measurement-limited
PLR	Cd-111	4023	+0.27% (-1.69% to +2.27%)	+0.04% (-0.25% to +0.33%)	0.789	0.866	Toxicological priority
PLR	As-75	4023	+0.15% (-0.64% to +0.95%)	+0.06% (-0.26% to +0.38%)	0.709	0.866	Toxicological priority
PLR	Cr-52	4023	-0.30% (-2.38% to +1.82%)	-0.03% (-0.24% to +0.18%)	0.775	0.866	Toxicological priority
PLR	Co-59	4023	+0.74% (+0.20% to +1.28%)	+0.96% (+0.26% to +1.67%)	0.008	0.064	Essential/homeostatic
PLR	Cu-63	4023	+1.55% (+0.44% to +2.68%)	+3.35% (+0.94% to +5.82%)	0.006	0.064	Essential/homeostatic
PLR	Fe-56	4023	-0.78% (-2.73% to +1.21%)	-0.96% (-3.37% to +1.50%)	0.439	0.847	Essential/homeostatic
PLR	Hg-202	3258	+1.58% (-1.06% to +4.30%)	+0.36% (-0.24% to +0.96%)	0.242	0.614	Measurement-limited
PLR	Mn-55	4023	+0.02% (-0.60% to +0.65%)	+0.02% (-0.43% to +0.47%)	0.947	0.965	Essential/homeostatic
PLR	Mo-98	4023	+0.91% (+0.24% to +1.59%)	+0.48% (+0.13% to +0.84%)	0.008	0.064	Essential/homeostatic
PLR	Ni-60	2213	+1.76% (-1.12% to +4.72%)	+0.25% (-0.16% to +0.65%)	0.231	0.614	Measurement-limited
PLR	Pb-208	4023	+0.29% (-0.73% to +1.32%)	+0.12% (-0.31% to +0.56%)	0.582	0.866	Measurement-limited
PLR	Sb-121	4023	+1.82% (-0.11% to +3.78%)	+0.25% (-0.02% to +0.52%)	0.065	0.274	Toxicological priority
PLR	Se-78	4023	+1.64% (+0.57% to +2.72%)	+3.14% (+1.08% to +5.25%)	0.003	0.055	Essential/homeostatic
PLR	Sr-88	4023	+0.26% (-0.94% to +1.48%)	+0.40% (-1.44% to +2.28%)	0.671	0.866	Other
PLR	Tl-205	4023	+0.81% (-1.28% to +2.95%)	+0.15% (-0.24% to +0.55%)	0.448	0.847	Toxicological priority
PLR	V-51	4023	-0.30% (-1.20% to +0.60%)	-0.18% (-0.71% to +0.35%)	0.508	0.847	Toxicological priority
PLR	Zn-66	4023	+0.08% (-0.82% to +0.99%)	+0.17% (-1.79% to +2.17%)	0.865	0.913	Essential/homeostatic

Laboratory outcome	Serum element	n	Per log-IQR, % (95% CI)	Per doubling, % (95% CI)	p	q	Class
RDW	Li-7	4023	-0.04% (-0.23% to +0.15%)	-0.04% (-0.23% to +0.14%)	0.648	0.866	Other
RDW	Be-9	4023	+0.06% (-0.22% to +0.33%)	+0.02% (-0.06% to +0.10%)	0.694	0.866	Measurement-limited
RDW	Cd-111	4023	+0.06% (-0.40% to +0.52%)	+0.01% (-0.06% to +0.08%)	0.798	0.866	Toxicological priority
RDW	As-75	4023	-0.03% (-0.21% to +0.15%)	-0.01% (-0.09% to +0.06%)	0.737	0.866	Toxicological priority
RDW	Cr-52	4023	-0.35% (-0.83% to +0.14%)	-0.04% (-0.08% to +0.01%)	0.159	0.465	Toxicological priority
RDW	Co-59	4023	+0.14% (-0.04% to +0.32%)	+0.18% (-0.05% to +0.42%)	0.130	0.437	Essential/homeostatic
RDW	Cu-63	4023	+0.18% (-0.16% to +0.52%)	+0.38% (-0.34% to +1.11%)	0.297	0.696	Essential/homeostatic
RDW	Fe-56	4023	-1.35% (-1.82% to -0.87%)	-1.66% (-2.24% to -1.08%)	8.92e-08	3.39e-06	Essential/homeostatic
RDW	Hg-202	3258	+0.17% (-0.44% to +0.78%)	+0.04% (-0.10% to +0.18%)	0.585	0.866	Measurement-limited
RDW	Mn-55	4023	-0.04% (-0.14% to +0.07%)	-0.03% (-0.10% to +0.05%)	0.476	0.847	Essential/homeostatic
RDW	Mo-98	4023	-0.12% (-0.28% to +0.04%)	-0.06% (-0.15% to +0.02%)	0.138	0.437	Essential/homeostatic
RDW	Ni-60	2213	-0.21% (-0.85% to +0.43%)	-0.03% (-0.12% to +0.06%)	0.513	0.847	Measurement-limited
RDW	Pb-208	4023	+0.10% (-0.10% to +0.30%)	+0.04% (-0.04% to +0.13%)	0.312	0.696	Measurement-limited
RDW	Sb-121	4023	-0.60% (-1.06% to -0.13%)	-0.08% (-0.15% to -0.02%)	0.013	0.081	Toxicological priority
RDW	Se-78	4023	+0.31% (+0.05% to +0.58%)	+0.60% (+0.10% to +1.10%)	0.020	0.106	Essential/homeostatic
RDW	Sr-88	4023	+0.30% (-0.01% to +0.60%)	+0.46% (-0.01% to +0.93%)	0.057	0.271	Other
RDW	Tl-205	4023	-0.10% (-0.54% to +0.34%)	-0.02% (-0.10% to +0.06%)	0.654	0.866	Toxicological priority
RDW	V-51	4023	-0.05% (-0.24% to +0.14%)	-0.03% (-0.14% to +0.08%)	0.621	0.866	Toxicological priority
RDW	Zn-66	4023	+0.17% (-0.18% to +0.52%)	+0.37% (-0.39% to +1.13%)	0.339	0.716	Essential/homeostatic

PLR and RDW formed one combined 38-test family. Log-IQR estimates represent element-specific distribution contrasts. Per-doubling estimates use a common relative concentration increment; neither scale establishes comparative biological potency. Algebraic rescaling leaves p and q values unchanged.

Supplementary Table S10. Complete Model C results for glucose and homocysteine.

Laboratory outcome	Serum element	n	Per log-IQR, % (95% CI)	Per doubling, % (95% CI)	p	q	Class
Glucose	Li-7	4023	+0.13% (-0.16% to +0.41%)	+0.12% (-0.16% to +0.41%)	0.391	0.629	Other
Glucose	Be-9	4023	-0.02% (-0.59% to +0.55%)	-0.01% (-0.17% to +0.16%)	0.942	0.987	Measurement-limited

Laboratory outcome	Serum element	n	Per log-IQR, % (95% CI)	Per doubling, % (95% CI)	p	q	Class
Glucose	Cd-111	4023	+0.12% (-0.64% to +0.87%)	+0.02% (-0.09% to +0.13%)	0.763	0.866	Toxicological priority
Glucose	As-75	4023	-0.12% (-0.46% to +0.22%)	-0.05% (-0.19% to +0.09%)	0.476	0.646	Toxicological priority
Glucose	Cr-52	4023	+0.11% (-0.67% to +0.90%)	+0.01% (-0.07% to +0.09%)	0.775	0.866	Toxicological priority
Glucose	Co-59	4023	-0.17% (-0.37% to +0.03%)	-0.22% (-0.48% to +0.04%)	0.090	0.335	Essential/homeostatic
Glucose	Cu-63	4023	-0.17% (-0.45% to +0.11%)	-0.36% (-0.96% to +0.24%)	0.237	0.563	Essential/homeostatic
Glucose	Fe-56	4023	-0.21% (-0.70% to +0.28%)	-0.26% (-0.87% to +0.35%)	0.394	0.629	Essential/homeostatic
Glucose	Hg-202	3258	-0.38% (-1.26% to +0.51%)	-0.09% (-0.29% to +0.11%)	0.397	0.629	Measurement-limited
Glucose	Mn-55	4023	-0.14% (-0.39% to +0.11%)	-0.10% (-0.28% to +0.08%)	0.269	0.567	Essential/homeostatic
Glucose	Mo-98	4023	-0.39% (-0.65% to -0.12%)	-0.21% (-0.35% to -0.06%)	0.005	0.093	Essential/homeostatic
Glucose	Ni-60	2213	+0.36% (-0.73% to +1.45%)	+0.05% (-0.10% to +0.20%)	0.521	0.660	Measurement-limited
Glucose	Pb-208	4023	-0.14% (-0.52% to +0.23%)	-0.06% (-0.22% to +0.10%)	0.446	0.646	Measurement-limited
Glucose	Sb-121	4023	+0.64% (-0.14% to +1.41%)	+0.09% (-0.02% to +0.20%)	0.106	0.335	Toxicological priority
Glucose	Se-78	4023	0.00% (-0.38% to +0.38%)	-0.01% (-0.73% to +0.72%)	0.987	0.987	Essential/homeostatic
Glucose	Sr-88	4023	+0.41% (-0.02% to +0.84%)	+0.62% (-0.03% to +1.29%)	0.063	0.299	Other
Glucose	Tl-205	4023	-0.83% (-1.65% to -0.01%)	-0.16% (-0.31% to 0.00%)	0.048	0.299	Toxicological priority
Glucose	V-51	4023	-0.34% (-0.68% to +0.01%)	-0.20% (-0.40% to +0.01%)	0.057	0.299	Toxicological priority
Glucose	Zn-66	4023	-0.21% (-0.52% to +0.10%)	-0.47% (-1.14% to +0.21%)	0.178	0.483	Essential/homeostatic
Homocysteine	Li-7	4023	-0.02% (-0.47% to +0.44%)	-0.02% (-0.47% to +0.44%)	0.938	0.983	Other
Homocysteine	Be-9	4023	-0.68% (-1.41% to +0.05%)	-0.19% (-0.40% to +0.02%)	0.069	0.284	Measurement-limited
Homocysteine	Cd-111	4023	+0.28% (-1.02% to +1.60%)	+0.04% (-0.15% to +0.23%)	0.671	0.907	Toxicological priority
Homocysteine	As-75	4023	-0.22% (-0.73% to +0.29%)	-0.09% (-0.30% to +0.12%)	0.391	0.646	Toxicological priority
Homocysteine	Cr-52	4023	-0.96% (-2.36% to +0.46%)	-0.10% (-0.24% to +0.05%)	0.181	0.493	Toxicological priority
Homocysteine	Co-59	4023	+0.09% (-0.53% to +0.72%)	+0.12% (-0.68% to +0.94%)	0.764	0.907	Essential/homeostatic
Homocysteine	Cu-63	4023	+0.10% (-0.44% to +0.65%)	+0.21% (-0.95% to +1.39%)	0.720	0.907	Essential/homeostatic
Homocysteine	Fe-56	4023	+0.71% (-0.07% to +1.50%)	+0.88% (-0.09% to +1.86%)	0.075	0.284	Essential/homeostatic

Laboratory outcome	Serum element	n	Per log-IQR, % (95% CI)	Per doubling, % (95% CI)	p	q	Class
Homocysteine	Hg-202	3258	-0.82% (-2.43% to +0.82%)	-0.19% (-0.56% to +0.19%)	0.324	0.646	Measurement-limited
Homocysteine	Mn-55	4023	-0.20% (-0.62% to +0.22%)	-0.15% (-0.45% to +0.16%)	0.346	0.646	Essential/homeostatic
Homocysteine	Mo-98	4023	+0.77% (+0.32% to +1.22%)	+0.41% (+0.17% to +0.65%)	9.48e-04	0.009	Essential/homeostatic
Homocysteine	Ni-60	2213	+3.36% (+1.63% to +5.12%)	+0.47% (+0.23% to +0.71%)	1.60e-04	0.003	Measurement-limited
Homocysteine	Pb-208	4023	+0.26% (-0.34% to +0.86%)	+0.11% (-0.14% to +0.37%)	0.391	0.646	Measurement-limited
Homocysteine	Sb-121	4023	+1.77% (+0.31% to +3.25%)	+0.25% (+0.04% to +0.45%)	0.018	0.114	Toxicological priority
Homocysteine	Se-78	4023	+0.08% (-0.80% to +0.96%)	+0.15% (-1.52% to +1.84%)	0.862	0.963	Essential/homeostatic
Homocysteine	Sr-88	4023	-0.19% (-0.82% to +0.46%)	-0.28% (-1.26% to +0.70%)	0.568	0.831	Other
Homocysteine	Tl-205	4023	+0.62% (-0.84% to +2.10%)	+0.12% (-0.16% to +0.39%)	0.408	0.646	Toxicological priority
Homocysteine	V-51	4023	-0.57% (-1.22% to +0.10%)	-0.33% (-0.72% to +0.06%)	0.094	0.299	Toxicological priority
Homocysteine	Zn-66	4023	+0.01% (-0.49% to +0.50%)	+0.01% (-1.07% to +1.11%)	0.983	0.983	Essential/homeostatic

Glucose and exploratory homocysteine each formed a separate 19-test family. Log-IQR estimates represent element-specific distribution contrasts. Per-doubling estimates use a common relative concentration increment; neither scale establishes comparative biological potency. Algebraic rescaling leaves p and q values unchanged.

Supplementary Table S11. Fe-57 analytical consistency channel.

Outcome	n	% difference (95% CI)	p
Serum glucose	4023	-0.15% (-0.65 to +0.36)	0.563
Homocysteine	4023	+0.76% (+0.25 to +1.28)	0.004
LDL-C	4023	+0.27% (-1.10 to +1.65)	0.702
NLR	4023	-0.40% (-3.07 to +2.35)	0.773
PLR	4023	-0.80% (-2.92 to +1.37)	0.465
RDW	4023	-1.20% (-1.73 to -0.66)	1.90e-05
SII	4023	-0.38% (-3.35 to +2.67)	0.802

Fe-57 was a duplicate isotope measurement of iron used for analytical consistency and was outside the 19-element primary FDR families. Fe-56 was the primary iron channel.

Supplementary Table S12. Restricted cubic spline tests.

Outcome	Element	p global	q global	p nonlinear	q nonlinear
LDL-C	Se-78	3.78e-05	6.43e-04	0.745	0.745
LDL-C	Cu-63	4.36e-04	0.004	0.438	0.621
LDL-C	Hg-202	0.002	0.009	0.533	0.647
LDL-C	Zn-66	0.002	0.009	0.048	0.423
LDL-C	As-75	0.058	0.198	0.659	0.736
LDL-C	Tl-205	0.074	0.209	0.058	0.423

Outcome	Element	p global	q global	p nonlinear	q nonlinear
LDL-C	Fe-56	0.116	0.263	0.259	0.591
LDL-C	Mo-98	0.124	0.263	0.173	0.591
LDL-C	Sr-88	0.164	0.310	0.075	0.423
LDL-C	Ni-60	0.217	0.370	0.278	0.591
LDL-C	Li-7	0.337	0.440	0.504	0.647
LDL-C	Cr-52	0.332	0.440	0.235	0.591
LDL-C	Sb-121	0.362	0.440	0.693	0.736
LDL-C	V-51	0.293	0.440	0.413	0.621
LDL-C	Mn-55	0.420	0.476	0.363	0.621
LDL-C	Cd-111	0.490	0.490	0.230	0.591
LDL-C	Co-59	0.473	0.490	0.383	0.621
NLR	Co-59	9.83e-04	0.017	0.151	0.348
NLR	As-75	0.006	0.053	0.006	0.094
NLR	Cr-52	0.040	0.223	0.184	0.348
NLR	Ni-60	0.053	0.223	0.534	0.757
NLR	Sb-121	0.067	0.227	0.386	0.596
NLR	Fe-56	0.139	0.263	0.110	0.348
NLR	Mn-55	0.121	0.263	0.817	0.886
NLR	Mo-98	0.139	0.263	0.767	0.886
NLR	Zn-66	0.126	0.263	0.183	0.348
NLR	V-51	0.174	0.296	0.099	0.348
NLR	Sr-88	0.209	0.322	0.258	0.439
NLR	Cd-111	0.292	0.354	0.151	0.348
NLR	Hg-202	0.277	0.354	0.112	0.348
NLR	Tl-205	0.263	0.354	0.122	0.348
NLR	Li-7	0.548	0.582	0.833	0.886
NLR	Se-78	0.539	0.582	0.766	0.886
NLR	Cu-63	0.824	0.824	0.890	0.890

Four separate 17-test BH families were used: LDL-C global association, LDL-C nonlinearity, NLR global association and NLR nonlinearity. These are secondary analyses. Be and Pb were excluded from spline families because their continuous lower tails were predominantly model-generated.

Supplementary Table S13. Selected robustness and native-unit analyses for FDR-significant or clinically salient findings.

Outcome	Serum element	Analysis	Difference (95% CI)	p	n	Inference
LDL-C	Hg-202	Main	+3.72% (+1.69 to +5.79)	3.47e-04	3258	Main family
		Direct quantified	+1.89% (-1.46 to +5.36)	0.269	2416	Nominal
		Quantification status	+4.08% (+1.77 to +6.43)	5.49e-04	3253	Nominal
		Strict sensitivity	+2.47% (+0.32 to +4.67)	0.025	2519	Nominal
		No reported dyslipidaemia	+3.12% (+1.07 to +5.22)	0.003	2784	Nominal
		Renal adjustment	+3.72% (+1.70 to +5.77)	3.27e-04	3258	Nominal

Outcome	Serum element	Analysis	Difference (95% CI)	p	n	Inference
		Municipality FE	+2.76% (+0.68 to +4.89)	0.010	3258	Nominal
		Occupational exclusion	+3.14% (+0.90 to +5.43)	0.006	2618	Nominal
		CR2	+3.72% (+1.66 to +5.82)	4.25e-04	3258	Nominal
		Assay IPW	+3.63% (+1.61 to +5.70)	4.66e-04	3258	Nominal
		Native-unit sensitivity	+4.35 mg/dL (+1.96 to +6.73)	4.19e-04	3258	Nominal
		Exclude current cholesterol medication	+3.49% (+1.51% to +5.51%)	5.74e-04	3207	Nominal
LDL-C	Se-78	Main	+2.04% (+1.14 to +2.95)	1.30e-05	4023	Main family
		Direct quantified	+2.37% (+1.46 to +3.28)	6.16e-07	4022	Nominal
		Strict sensitivity	+2.08% (+1.08 to +3.08)	5.09e-05	3117	Nominal
		No reported dyslipidaemia	+2.11% (+1.12 to +3.10)	3.50e-05	3443	Nominal
		Renal adjustment	+2.04% (+1.14 to +2.95)	1.34e-05	4023	Nominal
		Municipality FE	+2.12% (+1.18 to +3.07)	1.32e-05	4023	Nominal
		Occupational exclusion	+1.88% (+0.98 to +2.79)	5.34e-05	3221	Nominal
		CR2	+2.04% (+1.11 to +2.98)	2.52e-05	4023	Nominal
		Native-unit sensitivity	+2.55 mg/dL (+1.48 to +3.62)	5.00e-06	4023	Nominal
		Exclude current cholesterol medication	+2.18% (+1.20% to +3.16%)	1.69e-05	3957	Nominal
LDL-C	Cu-63	Main	+1.55% (+0.74 to +2.36)	2.14e-04	4023	Main family
		Direct quantified	+1.68% (+0.70 to +2.68)	8.55e-04	4019	Nominal
		Strict sensitivity	+1.56% (+0.60 to +2.54)	0.002	3117	Nominal
		No reported dyslipidaemia	+1.48% (+0.65 to +2.31)	5.40e-04	3443	Nominal
		Renal adjustment	+1.55% (+0.74 to +2.37)	2.02e-04	4023	Nominal
		Municipality FE	+1.57% (+0.80 to +2.35)	8.55e-05	4023	Nominal
		Occupational exclusion	+1.68% (+0.81 to +2.57)	1.99e-04	3221	Nominal
		CR2	+1.55% (+0.71 to +2.39)	3.50e-04	4023	Nominal
		Alternative Family-C completion	+1.55% (+0.75 to +2.37)	1.89e-04	4023	Nominal
		Native-unit sensitivity	+1.81 mg/dL (+0.88 to +2.73)	1.66e-04	4023	Nominal
		Exclude current cholesterol medication	+1.65% (+0.87% to +2.44%)	4.29e-05	3957	Nominal
LDL-C	Zn-66	Main	+1.26% (+0.49 to +2.04)	0.001	4023	Main family
		Direct quantified	+1.62% (+0.60 to +2.64)	0.002	3845	Nominal
		Strict sensitivity	+1.13% (+0.34 to +1.92)	0.005	3117	Nominal
		No reported dyslipidaemia	+1.25% (+0.49 to +2.02)	0.001	3443	Nominal
		Renal adjustment	+1.26% (+0.49 to +2.04)	0.001	4023	Nominal
		Municipality FE	+1.48% (+0.69 to +2.28)	2.92e-04	4023	Nominal

Outcome	Serum element	Analysis	Difference (95% CI)	p	n	Inference
		Occupational exclusion	+1.31% (+0.52 to +2.11)	0.001	3221	Nominal
		CR2	+1.26% (+0.48 to +2.05)	0.002	4023	Nominal
		Native-unit sensitivity	+1.41 mg/dL (+0.44 to +2.38)	0.005	4023	Nominal
		Exclude current cholesterol medication	+1.52% (+0.81% to +2.23%)	3.85e-05	3957	Nominal
SII	Sb-121	Main	+4.29% (+1.29 to +7.38)	0.005	4023	Main family
		Direct quantified	+0.93% (-8.50 to +11.33)	0.852	2563	Nominal
		Observed CBC	+4.20% (+1.21 to +7.27)	0.006	3938	Nominal
		Quantification status	+5.17% (+1.68 to +8.78)	0.004	4018	Nominal
		Strict sensitivity	+3.33% (-0.34 to +7.14)	0.076	3117	Nominal
		Alternative Family-C completion	+4.25% (+1.27 to +7.32)	0.005	4023	Nominal
SII	Cu-63	Main	+2.34% (+0.64 to +4.07)	0.007	4023	Main family
		Direct quantified	+2.94% (+1.02 to +4.89)	0.003	4019	Nominal
		Observed CBC	+2.36% (+0.67 to +4.08)	0.006	3938	Nominal
		Strict sensitivity	+1.48% (-0.40 to +3.38)	0.122	3117	Nominal
		Alternative Family-C completion	+2.33% (+0.66 to +4.04)	0.006	4023	Nominal
SII	Co-59	Main	+1.46% (+0.65 to +2.28)	4.89e-04	4023	Main family
		Direct quantified	+0.78% (-0.81 to +2.39)	0.336	3988	Nominal
		Observed CBC	+1.46% (+0.66 to +2.26)	3.59e-04	3938	Nominal
		Strict sensitivity	+1.25% (+0.43 to +2.08)	0.003	3117	Nominal
		Alternative Family-C completion	+1.43% (+0.63 to +2.24)	5.09e-04	4023	Nominal
SII	Mo-98	Main	+1.39% (+0.44 to +2.35)	0.004	4023	Main family
		Direct quantified	+2.54% (+0.10 to +5.04)	0.042	3427	Nominal
		Observed CBC	+1.37% (+0.44 to +2.30)	0.004	3938	Nominal
		Strict sensitivity	+1.51% (+0.45 to +2.58)	0.005	3117	Nominal
		Alternative Family-C completion	+1.31% (+0.38 to +2.25)	0.006	4023	Nominal
RDW	Fe-56	Main	-1.35% (-1.82 to -0.87)	8.92e-08	4023	Main family
		Direct quantified	-1.72% (-2.16 to -1.28)	1.99e-12	4012	Nominal
		Observed CBC	-1.35% (-1.83 to -0.87)	1.35e-07	3940	Nominal
		Strict sensitivity	-1.35% (-1.89 to -0.82)	1.80e-06	3117	Nominal
Homocysteine	Ni-60	Main	+3.36% (+1.63 to +5.12)	1.60e-04	2213	Main family
		Direct quantified	+6.16% (+0.02 to +12.68)	0.049	1536	Nominal
		Quantification status	+2.63% (+0.71 to +4.59)	0.007	2207	Nominal
		Strict sensitivity	+4.05% (+1.92 to +6.23)	2.14e-04	1684	Nominal

Outcome	Serum element	Analysis	Difference (95% CI)	p	n	Inference
		Renal adjustment	+3.29% (+1.52 to +5.09)	2.95e-04	2213	Nominal
		Assay availability IPW	+3.34% (+1.44% to +5.28%)	6.12e-04	2213	Nominal
Homocysteine	Mo-98	Main	+0.77% (+0.32 to +1.22)	9.48e-04	4023	Main family
		Direct quantified	+0.99% (-0.08 to +2.08)	0.069	3427	Nominal
		Strict sensitivity	+0.88% (+0.32 to +1.45)	0.002	3117	Nominal
		Renal adjustment	+0.75% (+0.28 to +1.21)	0.002	4023	Nominal
		Alternative Family-C completion	+0.76% (+0.32 to +1.21)	8.16e-04	4023	Nominal

Main estimates use their stated outcome-family FDR adjustment. Directly quantified concentration and quantification-status analyses were specified before association modelling and use nominal inference. The joint Se/Cu/Zn model, cholesterol medication exclusion and Ni–homocysteine assay availability weighting were post-primary exploratory analyses with nominal inference. Quantification status compares observed quantifiable and below-LOQ results; it does not estimate a continuous concentration gradient. Restrictions refer to the specified data component; these analyses are not interchangeable complete-case analyses. Blood-count outcomes may use completed components in directly quantified models, and serum predictors may use completed concentrations in observed-CBC models. Adjustment covariates retain the recorded implementation for each fitted analysis. Sensitivity p values are nominal; the original log-IQR reporting unit is retained for continuous contrasts. LDL-C native-unit sensitivity rows report coefficients from separate regressions of untransformed LDL-C in mg/dL per original element-specific log-IQR increase. They are not obtained by multiplying relative differences by the cohort median and are distinct from the standardized geometric-mean differences in Table S23. The corresponding percentage estimates use the same original log-IQR increment; their confidence intervals are shown in the respective rows. Sensitivity estimates and intervals use nominal inference. The table includes analyses specified before association modelling, diagnostics executed under predefined triggering rules and the post-primary exploratory additions identified in SM9 and the chronology. Their p values do not represent additional FDR-controlled discoveries; interpretation concerns magnitude, direction and uncertainty.

Supplementary Table S14. Highest FMI values within the two primary outcome families.

Outcome	Element	FMI	Relative efficiency	MCSE/SE	High FMI	High MCSE
LDL-C	Be-9	0.142	0.9953	0.065	False	False
NLR	Be-9	0.104	0.9965	0.054	False	False
NLR	Cd-111	0.082	0.9973	0.048	False	False
LDL-C	Zn-66	0.075	0.9975	0.045	False	False
NLR	Pb-208	0.073	0.9976	0.044	False	False
NLR	Mn-55	0.067	0.9978	0.042	False	False
LDL-C	Sb-121	0.058	0.9981	0.039	False	False
LDL-C	Tl-205	0.056	0.9981	0.038	False	False
NLR	Co-59	0.054	0.9982	0.037	False	False
NLR	Cr-52	0.053	0.9982	0.037	False	False
NLR	Ni-60	0.053	0.9982	0.036	False	False
NLR	Zn-66	0.050	0.9983	0.035	False	False
NLR	As-75	0.050	0.9983	0.035	False	False
LDL-C	Cd-111	0.049	0.9984	0.035	False	False
NLR	Mo-98	0.048	0.9984	0.034	False	False

The table shows the 15 highest FMI values within the NLR and LDL-C families. No primary coefficient exceeded the high-FMI or high-MCSE threshold. The maximum FMI across all Model C analyses was 0.177 (Be-9 and homocysteine; exploratory). FMI does not establish compatibility between inherited imputations and the substantive models.

Supplementary Table S15. Largest median inter-element Spearman correlations.

Element 1	Element 2	Median rho
Se-78	Zn-66	0.483
Li-7	Sr-88	0.411
Mn-55	Sb-121	0.371
Zn-66	Fe-56	0.358
Mo-98	Sb-121	0.358
Fe-56	Se-78	0.351
Cu-63	Se-78	0.333
Mn-55	Mo-98	0.327
Cr-52	Mn-55	0.325
Sr-88	Se-78	0.316

Supplementary Table S16. Largest standardized differences by Hg/Ni assay availability.

Element	Variable	Standardized difference
Ni-60	Impact area	0.452
Ni-60	Municipal cluster	0.436
Hg-202	Impact area	0.364
Hg-202	Municipal cluster	0.364
Ni-60	Questionnaire responder status	0.202
Ni-60	Smoking status	0.202
Hg-202	Smoking status	0.151
Hg-202	Questionnaire responder status	0.151
Hg-202	Homocysteine	0.138
Ni-60	Homocysteine	0.106
Ni-60	Age	0.076
Hg-202	LDL-C	-0.075
Ni-60	LDL-C	-0.063
Hg-202	Sex	0.054
Ni-60	Glucose	0.040

The largest standardized differences concerned environmental pressure area and municipal cluster (Ni: 0.452 and 0.436; Hg: 0.364 and 0.364). These observed patterns do not establish the operational cause of assay nonperformance. Inverse-probability weighting can address only selection explained by its measured covariates; residual selection associated with unrecorded laboratory batch, calendar time, specimen availability or other factors may remain. These comparisons are descriptive and do not demonstrate that weighting removed selection bias.

Supplementary Table S17. Influence diagnostics for FDR-significant LDL-C associations.

Element	Main %	Trimmed %	Robust %	n excluded	Max relative change	Influence-sensitive
Cu-63	+1.55%	+2.06%	+2.06%	42	32.9%	Yes
Hg-202	+3.72%	+3.65%	+3.68%	36	1.9%	No
Se-78	+2.04%	+2.44%	+2.53%	42	23.6%	No
Zn-66	+1.26%	+1.89%	+2.05%	42	62.4%	Yes

Main, trimmed and robust values are percentage differences in LDL-C per one-log-IQR increase in serum element concentration. Trimming excluded the 0.5% lower and 0.5% upper tails. Robust values are means across

imputation-specific M-estimates. Influence sensitivity was defined as a direction reversal or a maximum relative change greater than 25%. No direction reversal occurred.

Supplementary Table S18. Linked parent-questionnaire medication information.

Descriptor	Primary cohort (n=4,026)	All questionnaire responders (n=4,137)
Matched to parent questionnaire	4,026/4,026 (100%)	4,137/4,137 (100%)
Reported physician-diagnosed hypertension	444/4,026 (11.0%)	466/4,137 (11.3%)
Current medication for high blood pressure among those diagnosed	180/444 (40.5%)	190/466 (40.8%)
Identified current high-blood-pressure medication users / full cohort	180/4,026 (4.5%)	190/4,137 (4.6%)
Reported high cholesterol or triglycerides	580/4,026 (14.4%)	608/4,137 (14.7%)
Current cholesterol medication among those diagnosed	66/580 (11.4%)	72/608 (11.8%)
Identified current cholesterol medication users / full cohort	66/4,026 (1.6%)	72/4,137 (1.7%)
Reported diabetes	32/4,026 (0.8%)	35/4,137 (0.8%)
Insulin or other diabetes medication among those diagnosed	22/32 (68.8%)	22/35 (62.9%)
Identified insulin or other diabetes medication users / full cohort	22/4,026 (0.5%)	22/4,137 (0.5%)

For the diagnosis-linked follow-up questions, the identified users numbered 180 for current high-blood-pressure medication, 66 for current cholesterol medication and 22 for insulin or another diabetes medication, among diagnosis groups of 444, 580 and 32 participants, respectively. Missing follow-up responses were not interpreted as non-use. The counts do not establish complete ascertainment of treatment at blood collection. Medication questions were linked from the parent questionnaire by participant identifier. The variables were not regression adjustment covariates. “Current cholesterol medication” follows the questionnaire wording and does not establish a verified statin classification.

Supplementary Table S19. Element-specific log-IQR increments and original-unit serum concentration contrasts.

Q1 and Q3 are rounded primary-cohort serum concentration summaries in the original laboratory units. For channels with at least 25% left-censoring, Q1 was below the analytical LOQ and is shown as <LOQ; the completed-distribution fold equivalent is derived from the fixed completed-data log-IQR. These values aid interpretation and do not alter the fitted models.

Panel A. Original-unit concentration contrasts.

Element channel	Unit	Q1 concentration	Q3 concentration	Completed-distribution fold equivalent	Scaling-set n
Li-7	µg/L	0.736	1.49	2.02	4026
Be-9	µg/L	<0.0015	0.0123	11.4	4026
Cd-111	µg/L	<0.0010	0.0624	109	4026
As-75	µg/L	0.623	3.41	5.48	4026
Cr-52	µg/L	<0.0015	0.871	936	4026
Co-59	µg/L	0.712	1.21	1.71	4026
Cu-63	µg/L	842	1,164	1.38	4026
Fe-56	mg/L	0.903	1.58	1.75	4026
Hg-202	µg/L	<0.119	2.52	21.3	3259
Mn-55	µg/L	0.616	1.62	2.63	4026
Mo-98	µg/L	0.335	1.23	3.66	4026
Ni-60	µg/L	<0.0212	2.69	134	2213
Pb-208	µg/L	<0.0182	0.0565	5.07	4026
Sb-121	µg/L	<0.0020	0.192	138	4026
Se-78	µg/L	161	232	1.44	4026
Sr-88	µg/L	22.6	35.6	1.57	4026
Tl-205	µg/L	<0.0012	0.0462	38.7	4026
V-51	µg/L	0.216	0.703	3.26	4026
Zn-66	mg/L	1.22	1.66	1.37	4026
Fe-57	mg/L	0.891	1.58	1.77	4026

Panel B. Scaling constants and interpretive classification.

Element channel	Log-IQR	Interpretive class
Li-7	0.702	Other
Be-9	2.432	Measurement-limited
Cd-111	4.690	Toxicological priority
As-75	1.700	Toxicological priority
Cr-52	6.842	Toxicological priority
Co-59	0.534	Essential/homeostatic
Cu-63	0.324	Essential/homeostatic
Fe-56	0.560	Essential/homeostatic
Hg-202	3.059	Measurement-limited
Mn-55	0.965	Essential/homeostatic
Mo-98	1.298	Essential/homeostatic
Ni-60	4.897	Measurement-limited
Pb-208	1.622	Measurement-limited
Sb-121	4.930	Toxicological priority
Se-78	0.363	Essential/homeostatic
Sr-88	0.451	Other
Tl-205	3.656	Toxicological priority
V-51	1.181	Toxicological priority
Zn-66	0.317	Essential/homeostatic
Fe-57	0.571	Consistency channel

The log-IQR is the fixed median across 30 completed datasets of the element-specific IQR of natural-log concentration. The fold equivalent is exp(log-IQR), i.e., the Q3/Q1 concentration ratio represented by a one-unit model contrast. Large differences in fold equivalents mean that log-IQR effect magnitudes are not comparative

potency estimates; Tables S6–S10 also report common two-fold translations. Log-IQR estimates represent element-specific distribution contrasts. Per-doubling estimates use a common relative concentration increment; neither scale establishes comparative biological potency. Algebraic rescaling leaves p and q values unchanged.

Supplementary Table S20. Mutually adjusted Se-78, Cu-63 and Zn-66 model for LDL-C.

Term	n	Per log-IQR, % (95% CI)	Per doubling, % (95% CI)	p
Se-78	4023	+1.56% (+0.47% to +2.66%)	+2.99% (+0.89% to +5.13%)	0.005
Cu-63	4023	+0.87% (-0.15% to +1.91%)	+1.88% (-0.33% to +4.14%)	0.095
Zn-66	4023	+0.11% (-0.96% to +1.19%)	+0.24% (-2.09% to +2.63%)	0.839
Global 3-df test	4023	F=9.30	df=3, 173	9.87e-06

The joint model included Se-78, Cu-63 and Zn-66 simultaneously; age and BMI restricted cubic splines; sex; municipal-cluster fixed effects; smoking status; log1p alcohol, physical activity and energy intake; deprivation score; occupational exposure; and dietary zinc. The sample size was 4,023. Element-specific coefficients retain the original fixed log-IQR scaling, with algebraic per-doubling translations. The global test evaluates the three-element block. This was a post-primary exploratory sensitivity analysis with nominal inference and was not included in a primary FDR family. Individual Se and Cu models omitted dietary zinc; their differences from the joint estimates therefore cannot be attributed solely to mutual adjustment. The coefficients describe conditional associations, not independent causal effects.

Supplementary Table S21. LDL-C models excluding participants reporting current cholesterol medication.

Element	n	Per log-IQR, % (95% CI)	Per doubling, % (95% CI)	p
Li-7	3957	-0.46% (-1.17% to +0.26%)	-0.45% (-1.16% to +0.25%)	0.207
Be-9	3957	-0.56% (-1.76% to +0.66%)	-0.16% (-0.50% to +0.19%)	0.367
Cd-111	3957	+0.14% (-1.50% to +1.81%)	+0.02% (-0.22% to +0.27%)	0.864
As-75	3957	+0.79% (+0.10% to +1.49%)	+0.32% (+0.04% to +0.60%)	0.024
Cr-52	3957	-0.64% (-2.21% to +0.95%)	-0.07% (-0.23% to +0.10%)	0.424
Co-59	3957	-0.09% (-0.62% to +0.45%)	-0.11% (-0.80% to +0.59%)	0.753
Cu-63	3957	+1.65% (+0.87% to +2.44%)	+3.57% (+1.88% to +5.29%)	4.29e-05
Fe-56	3957	+0.62% (-0.46% to +1.72%)	+0.77% (-0.57% to +2.13%)	0.258
Hg-202	3207	+3.49% (+1.51% to +5.51%)	+0.78% (+0.34% to +1.22%)	5.74e-04
Mn-55	3957	-0.27% (-0.84% to +0.30%)	-0.19% (-0.60% to +0.21%)	0.348
Mo-98	3957	-0.43% (-1.26% to +0.40%)	-0.23% (-0.68% to +0.21%)	0.306
Ni-60	2175	-2.29% (-4.84% to +0.34%)	-0.33% (-0.70% to +0.05%)	0.087
Pb-208	3957	-0.92% (-1.75% to -0.09%)	-0.40% (-0.75% to -0.04%)	0.031
Sb-121	3957	+1.37% (-0.36% to +3.12%)	+0.19% (-0.05% to +0.43%)	0.120
Se-78	3957	+2.18% (+1.20% to +3.16%)	+4.19% (+2.30% to +6.11%)	1.69e-05
Sr-88	3957	-0.40% (-1.54% to +0.75%)	-0.62% (-2.35% to +1.15%)	0.488
Tl-205	3957	+1.48% (-0.53% to +3.53%)	+0.28% (-0.10% to +0.66%)	0.149
V-51	3957	-0.48% (-1.31% to +0.36%)	-0.28% (-0.77% to +0.21%)	0.260
Zn-66	3957	+1.52% (+0.81% to +2.23%)	+3.35% (+1.78% to +4.94%)	3.85e-05

Sixty-six participants identified by the current cholesterol medication question were excluded. Models otherwise used the Model C covariate set and the original element-specific scaling constants. This was a post-primary exploratory sensitivity analysis with nominal inference. It assesses the influence of identified current cholesterol medication users and does not exclude residual medication confounding.

Supplementary Table S22. Ni-60–homocysteine association after inverse-probability weighting for assay availability.

Analysis	n	Effective n	Effect per log-IQR (95% CI)	Effect per doubling (95% CI)	p	Weight range
Ni-60 and homocysteine assay-IPW	2213	1991	+3.34% (+1.44% to +5.28%)	+0.47% (+0.20% to +0.73%)	6.12e-04	0.56-2.28

Stabilized assay availability weights were truncated at the 1st and 99th percentiles. This post-primary exploratory sensitivity analysis extended the original Hg/Ni assay availability weighting programme to the Ni–homocysteine outcome and used nominal inference. Weighting addresses selection explained by the observed covariates in the weighting model but cannot eliminate selection related to unrecorded laboratory batch, calendar time, specimen availability or other factors. Similarity to the primary estimate does not establish absence of assay-selection bias.

Supplementary Table S23. Model-standardized geometric means at illustrative serum trace-element contrasts.

Values are covariate-standardized geometric means from Model C, pooled across 30 completed datasets. Panel A uses one fixed log-IQR anchored at reconstructed Q1; Panel B compares below-LOQ with quantifiable concentrations among assay-performed participants. Concentration endpoints are approximate. The fixed log-IQR contrasts can differ slightly from the approximate Q1–Q3 intervals in Table S19. The calculation is described in SM6.

Serum element concentration contrast	Laboratory outcome	Adjusted outcome: lower → higher (95% CI)	Absolute difference (95% CI)	Relative difference (95% CI)	p	Matching model q
Panel A. Continuous concentration contrasts: one fixed log-IQR, approximately equivalent to Q1–Q3.						
Se-78: 161 to 232 µg/L	LDL-C	113.2 (111.9–114.5) → 115.5 (114.0–117.0) mg/dL	+2.3 (+1.3 to +3.3) mg/dL	+2.04% (+1.14% to +2.95%)	1.30e-05	2.47e-04
Cu-63: 842 to 1,164 µg/L	LDL-C	113.4 (112.0–114.7) → 115.1 (113.7–116.5) mg/dL	+1.8 (+0.8 to +2.7) mg/dL	+1.55% (+0.74% to +2.36%)	2.14e-04	0.002
Zn-66: 1.22 to 1.67 mg/L	LDL-C	113.6 (112.3–114.9) → 115.0 (113.6–116.5) mg/dL	+1.4 (+0.6 to +2.3) mg/dL	+1.26% (+0.49% to +2.04%)	0.001	0.007
Co-59: 0.712 to 1.21 µg/L	SII	364.9 (359.2–370.7) → 370.3 (364.3–376.3) index units	+5.3 (+2.4 to +8.3) index units	+1.46% (+0.65% to +2.28%)	4.89e-04	0.009
Mo-98: 0.335 to 1.23 µg/L	SII	366.3 (360.8–372.0) → 371.4 (364.9–378.1) index units	+5.1 (+1.6 to +8.6) index units	+1.39% (+0.44% to +2.35%)	0.004	0.032
Cu-63: 842 to 1,164 µg/L	SII	362.6 (356.3–369.1) → 371.1 (364.6–377.8) index units	+8.5 (+2.3 to +14.6) index units	+2.34% (+0.64% to +4.07%)	0.007	0.034
Fe-56: 0.902 to 1.58 mg/L	RDW	12.28 (12.24–12.32) → 12.11 (12.07–12.16) %	−0.17 (−0.22 to −0.11) percentage points	−1.35% (−1.82% to −0.87%)	8.92e-08	3.39e-06
Mo-98: 0.335 to 1.23 µg/L	Homocysteine	8.23 (8.16–8.30) → 8.29 (8.21–8.37) µmol/L	+0.06 (+0.03 to +0.10) µmol/L	+0.77% (+0.32% to +1.22%)	9.48e-04	0.009
Panel B. Quantification-status contrasts among assay-performed participants						
Hg-202: below LOQ (<0.1192 µg/L) versus quantifiable (≥LOQ)	LDL-C	110.4 (108.2–112.7) → 114.9 (113.6–116.3) mg/dL	+4.5 (+2.0 to +7.0) mg/dL	+4.08% (+1.77% to +6.43%)	5.49e-04	—
Sb-121: below LOQ (<0.002 µg/L) versus quantifiable (≥LOQ)	SII	355.1 (345.8–364.6) → 373.4 (366.3–380.7) index units	+18.4 (+6.2 to +30.6) index units	+5.17% (+1.68% to +8.78%)	0.004	—
Ni-60: below LOQ (<0.0212 µg/L) versus quantifiable (≥LOQ)	Homocysteine	8.12 (8.00–8.25) → 8.34 (8.24–8.43) µmol/L	+0.21 (+0.06 to +0.37) µmol/L	+2.63% (+0.71% to +4.59%)	0.007	—

Values summarize conditional associations across the relevant analysis cohort; they do not estimate what would happen to an individual after a concentration change and do not define causal intervention effects, biological cut-offs or treatment targets. Confidence intervals describe uncertainty in standardized means or differences, not individual prediction intervals. Absolute differences concern the displayed geometric means and are distinct from native-outcome regression coefficients in Table S13. Relative and absolute differences within each row use the same exposure contrast. Panel A retains the p and q values from the matching continuous main model. Panel B reports nominal p values for the binary quantification-status models; no continuous-model q value is transferred

to these contrasts. For Hg–LDL-C, quantification status and LDL-C were observed, and this contrast does not require numerical imputation of below-LOQ element concentrations. This does not imply that every adjustment covariate or every blood-count component in the other models was observed. For RDW, the absolute difference and its confidence interval are expressed in percentage points; the relative difference is expressed as a percentage. Illustrative LDL-C and SII geometric means are displayed to one decimal place; RDW and homocysteine geometric means are displayed to two decimal places. Concentration intervals are rounded for readability; absolute differences and confidence intervals are calculated from unrounded predictions. Small discrepancies between displayed endpoint differences and reported contrasts may therefore arise from rounding.

Supplementary Table S24. Additional revision-stage multiplicity assessment across the 38 co-primary Model C comparisons.

Outcome	Serum element	Original p	Within-outcome q	Combined BH38 q	Original q < 0.05	Combined q < 0.05
LDL-C	As	0.0171579	0.0652002	0.0931431	No	No
LDL-C	Be	0.353205	0.412268	0.47935	No	No
LDL-C	Cd	0.94405	0.94405	0.94405	No	No
LDL-C	Co	0.518711	0.547529	0.607099	No	No
LDL-C	Cr	0.368872	0.412268	0.483349	No	No
LDL-C	Cu	2.14269e-04	0.00203556	0.00407112	Yes	Yes
LDL-C	Fe	0.257271	0.376011	0.444377	No	No
LDL-C	Hg	3.46833e-04	0.00219661	0.00439322	Yes	Yes
LDL-C	Li	0.156682	0.330773	0.350231	No	No
LDL-C	Mn	0.328488	0.412268	0.462316	No	No
LDL-C	Mo	0.191029	0.362954	0.382057	No	No
LDL-C	Ni	0.0986641	0.267802	0.267802	No	No
LDL-C	Pb	0.0439237	0.139092	0.1439	No	No
LDL-C	Sb	0.137137	0.3257	0.32892	No	No
LDL-C	Se	1.30174e-05	2.4733e-04	4.9466e-04	Yes	Yes
LDL-C	Sr	0.327465	0.412268	0.462316	No	No
LDL-C	Tl	0.251637	0.376011	0.444377	No	No
LDL-C	V	0.22853	0.376011	0.434207	No	No
LDL-C	Zn	0.00145481	0.00691036	0.0138207	Yes	Yes
NLR	As	0.166441	0.351375	0.351375	No	No
NLR	Be	0.394177	0.576105	0.499291	No	No
NLR	Cd	0.543194	0.645043	0.607099	No	No
NLR	Co	0.00762027	0.108987	0.057914	No	No
NLR	Cr	0.0141668	0.108987	0.0897228	No	No
NLR	Cu	0.539371	0.645043	0.607099	No	No
NLR	Fe	0.768087	0.810758	0.810758	No	No
NLR	Hg	0.900901	0.900901	0.92525	No	No
NLR	Li	0.311095	0.492567	0.462316	No	No
NLR	Mn	0.0348463	0.132416	0.132416	No	No
NLR	Mo	0.0454423	0.1439	0.1439	No	No
NLR	Ni	0.0200068	0.108987	0.0950323	No	No
NLR	Pb	0.473642	0.642799	0.580593	No	No
NLR	Sb	0.0229447	0.108987	0.0968777	No	No
NLR	Se	0.280104	0.492567	0.462316	No	No
NLR	Sr	0.138493	0.32892	0.32892	No	No
NLR	Tl	0.646559	0.722625	0.701978	No	No
NLR	V	0.29836	0.492567	0.462316	No	No
NLR	Zn	0.0930409	0.252539	0.267802	No	No

BH, Benjamini–Hochberg. Each original outcome family comprised 19 comparisons; the combined family comprised 38. Calculations used the original unrounded p values; numerical values displayed here are rounded to six significant figures. Four LDL-C associations and no NLR associations met the combined q < 0.05 threshold. The original within-outcome q values apply to their specified families, not to all analyses in the study.

Supplementary Figures

Supplementary Figure S1. Cross-outcome effect heatmap.

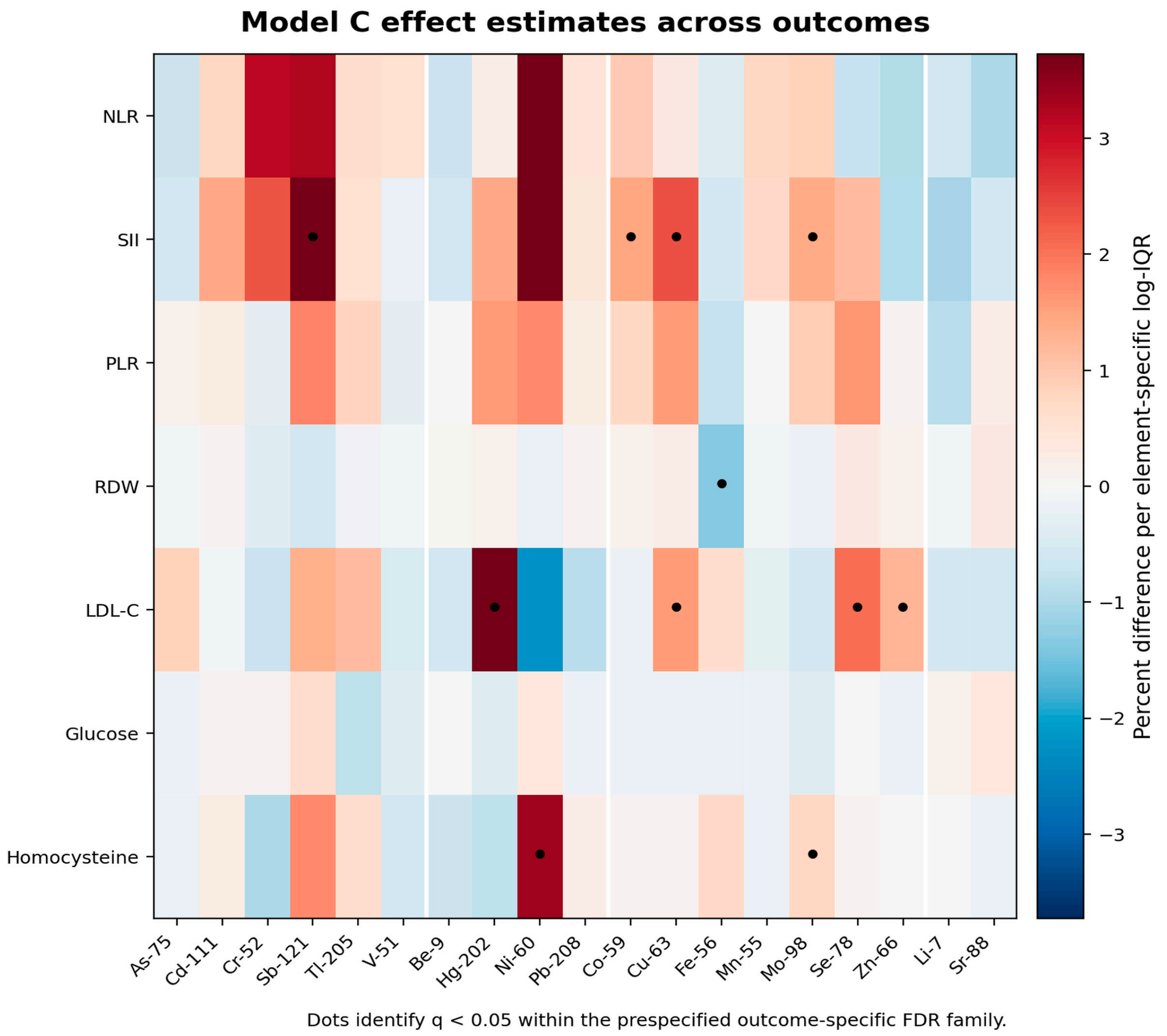

Model C percentage differences in laboratory outcomes per one element-specific log-IQR increase in serum element concentration across all specified outcomes. Rows represent laboratory outcomes and columns represent serum elements. Cells show percentage differences per element-specific log-IQR increase. The underlying concentration increment differs between elements, so colour intensity should not be interpreted as comparative potency; common per-doubling estimates are available in Tables S6–S10.

Supplementary Figure S2. Serum element correlation heatmap.

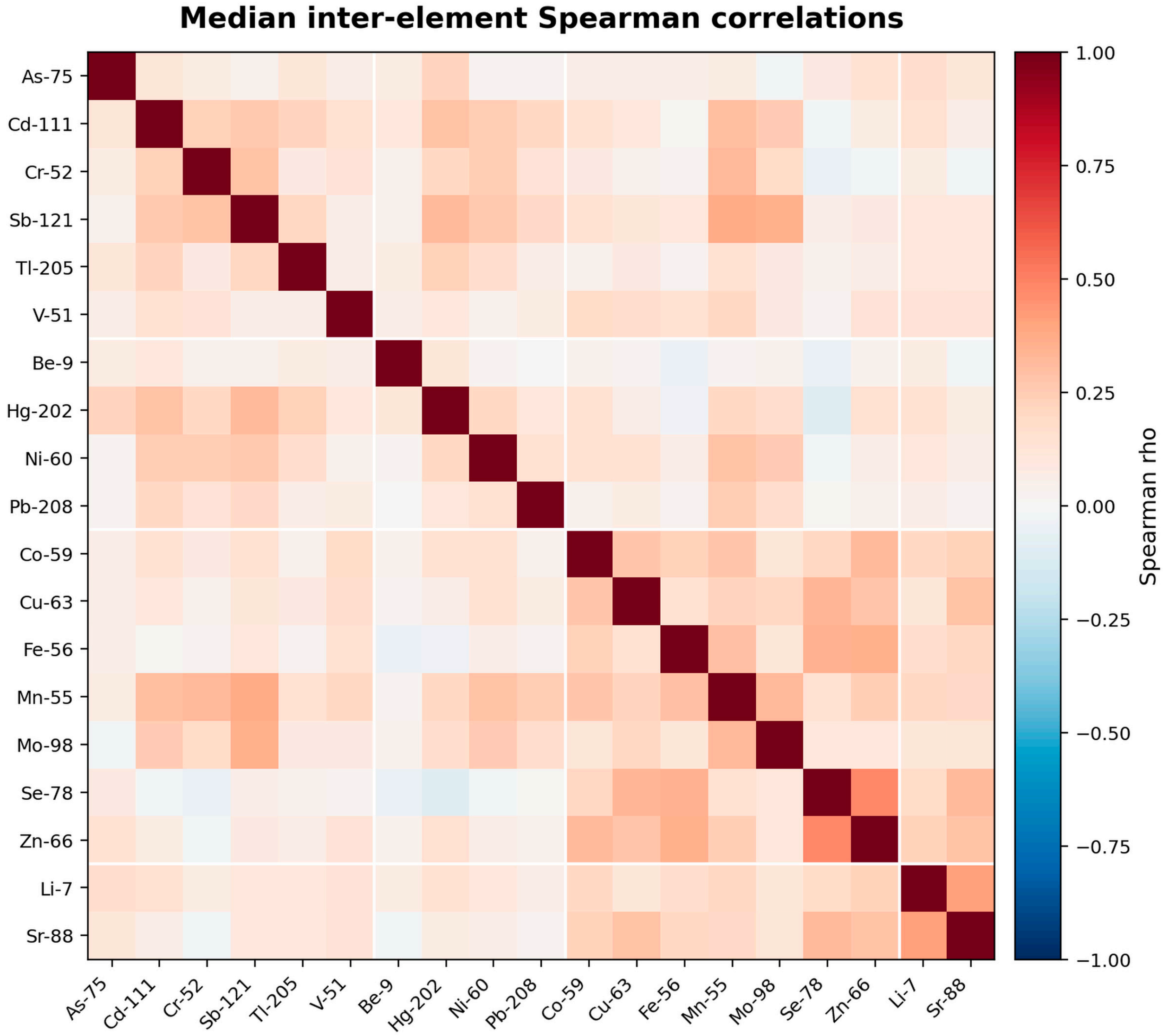

Median Spearman correlations among serum trace-element concentrations across the 30 completed datasets in the primary cohort.

Supplementary Figure S3. Hg-202 and LDL-C spline.

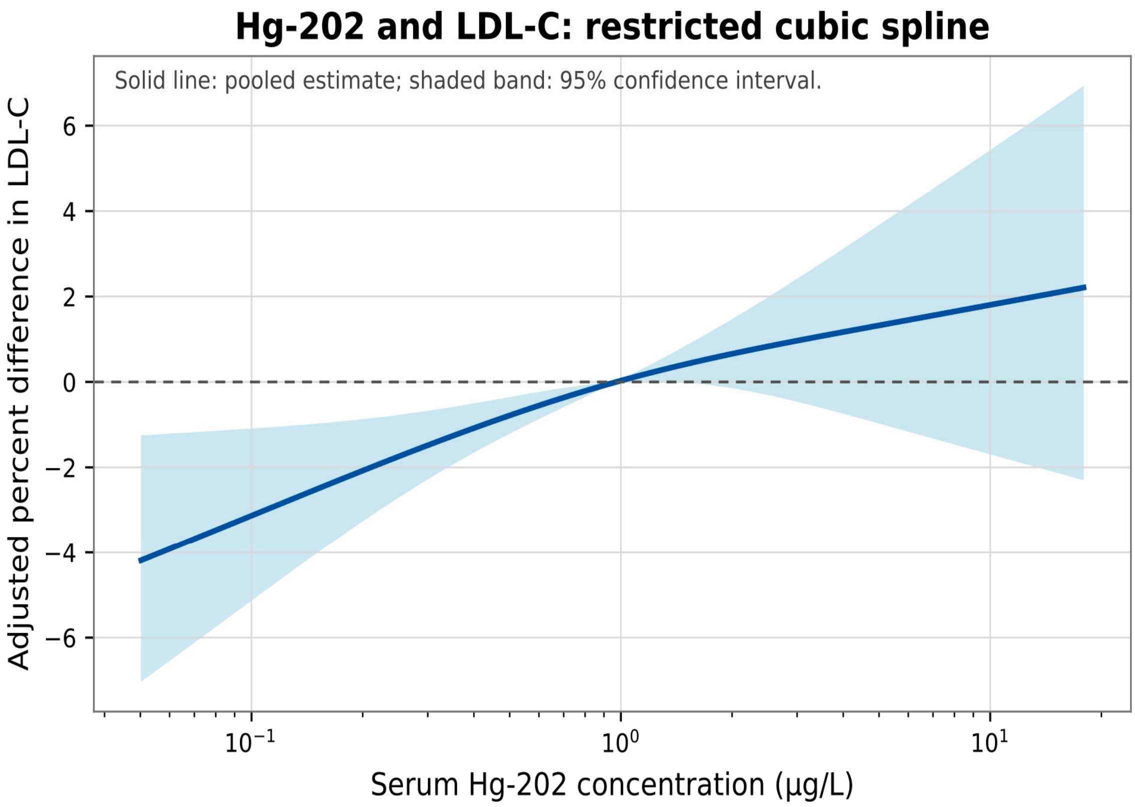

Restricted cubic spline from the fully adjusted model. The global association was FDR-significant, whereas the test for nonlinearity was not statistically significant.

Supplementary Figure S4. Se-78 and LDL-C spline.

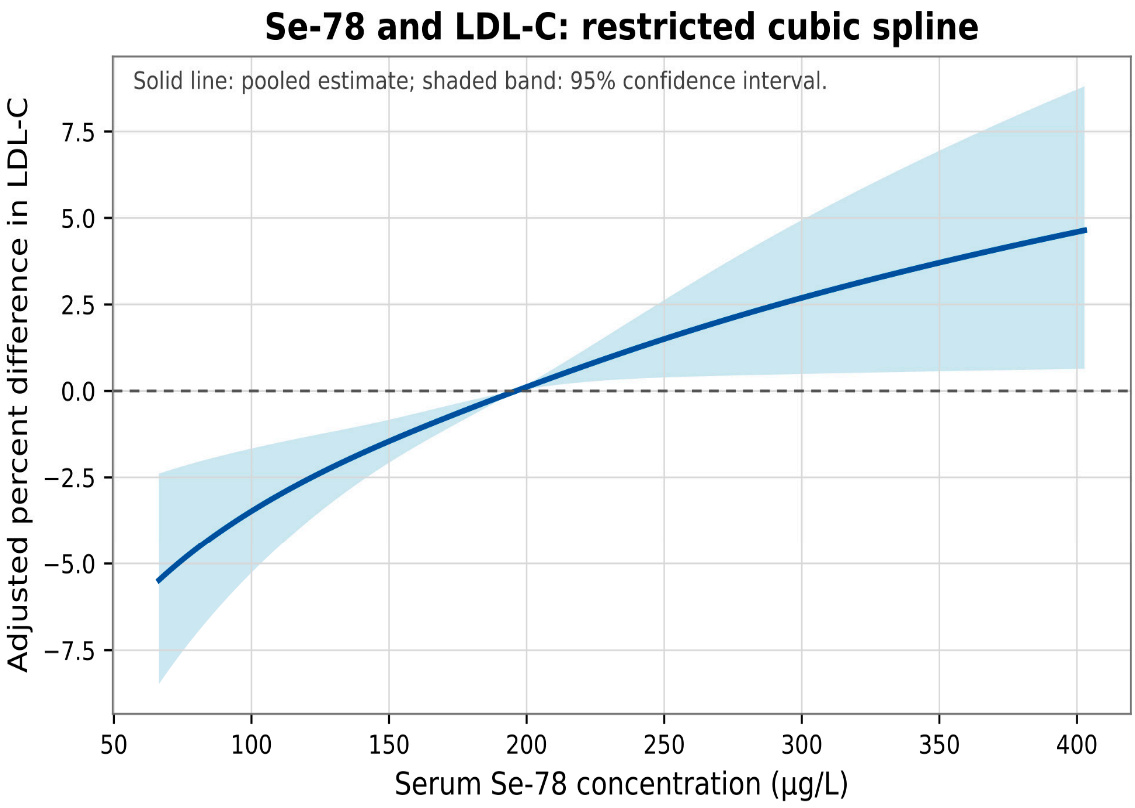

Restricted cubic spline from the fully adjusted model.

Supplementary Figure S5. Cu-63 and LDL-C spline.

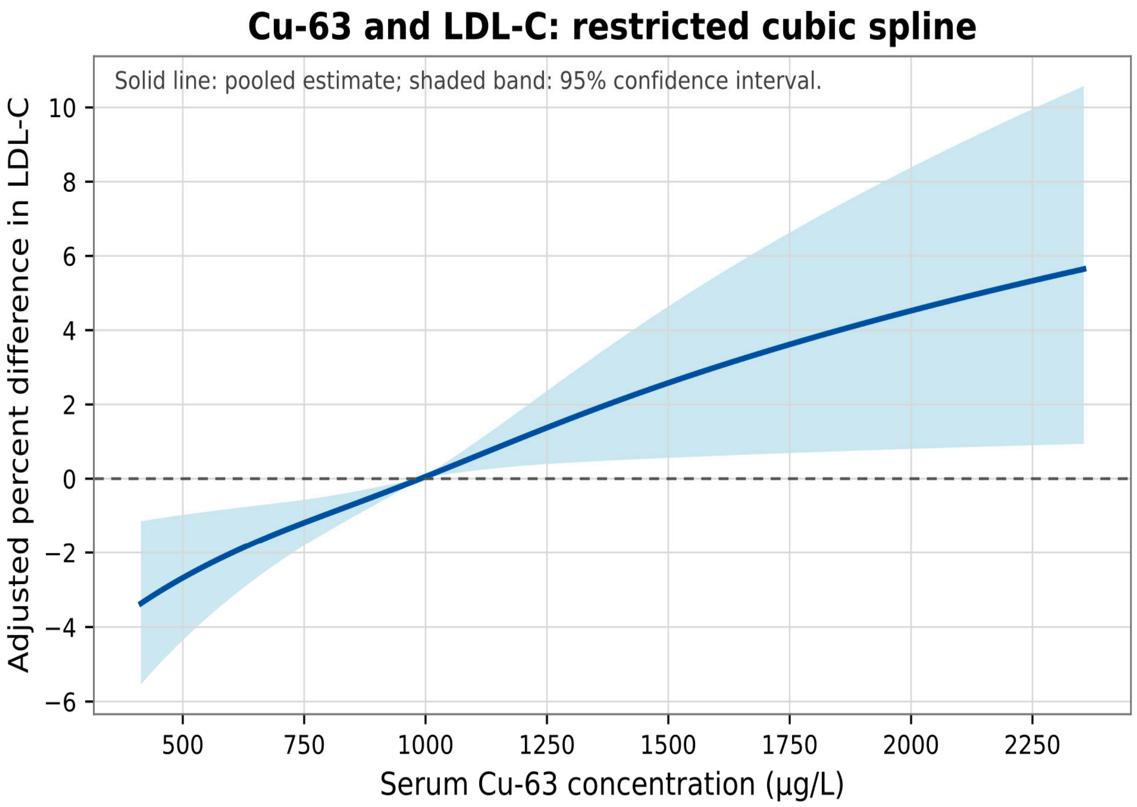

Restricted cubic spline from the fully adjusted model.

Supplementary Figure S6. Zn-66 and LDL-C spline.

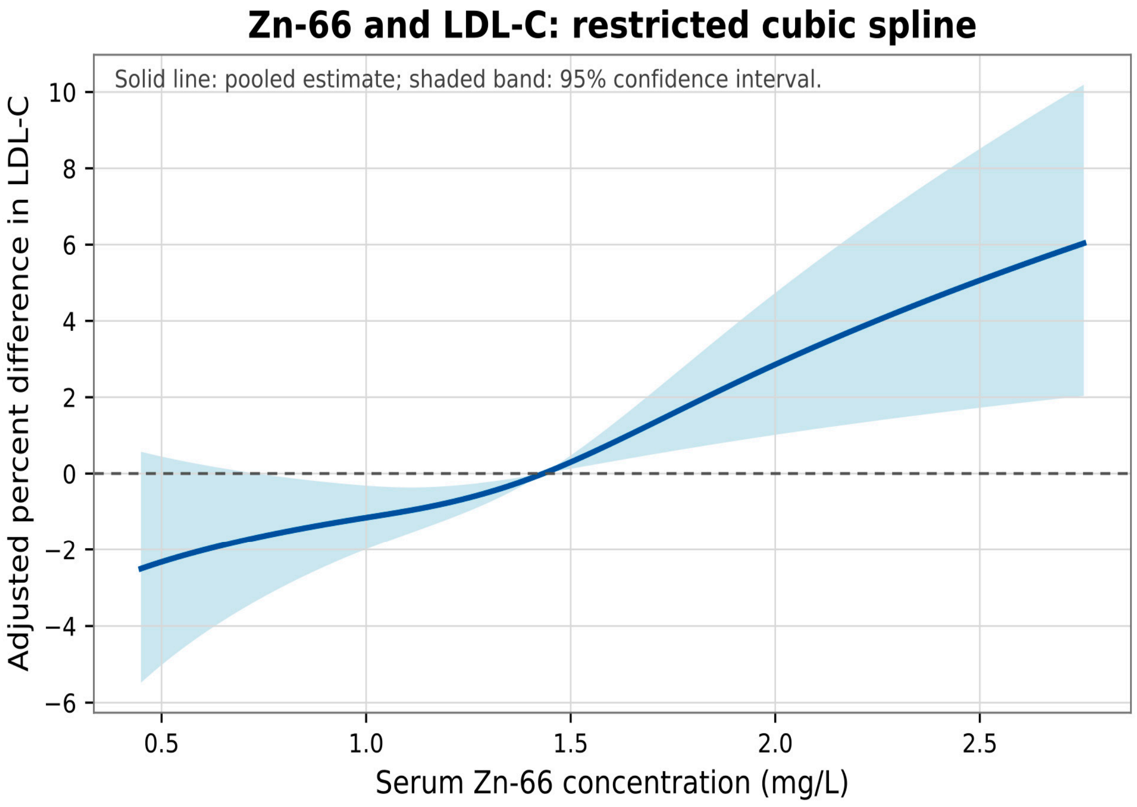

Restricted cubic spline from the fully adjusted model.

Supplementary Figure S7. Co-59 and NLR spline.

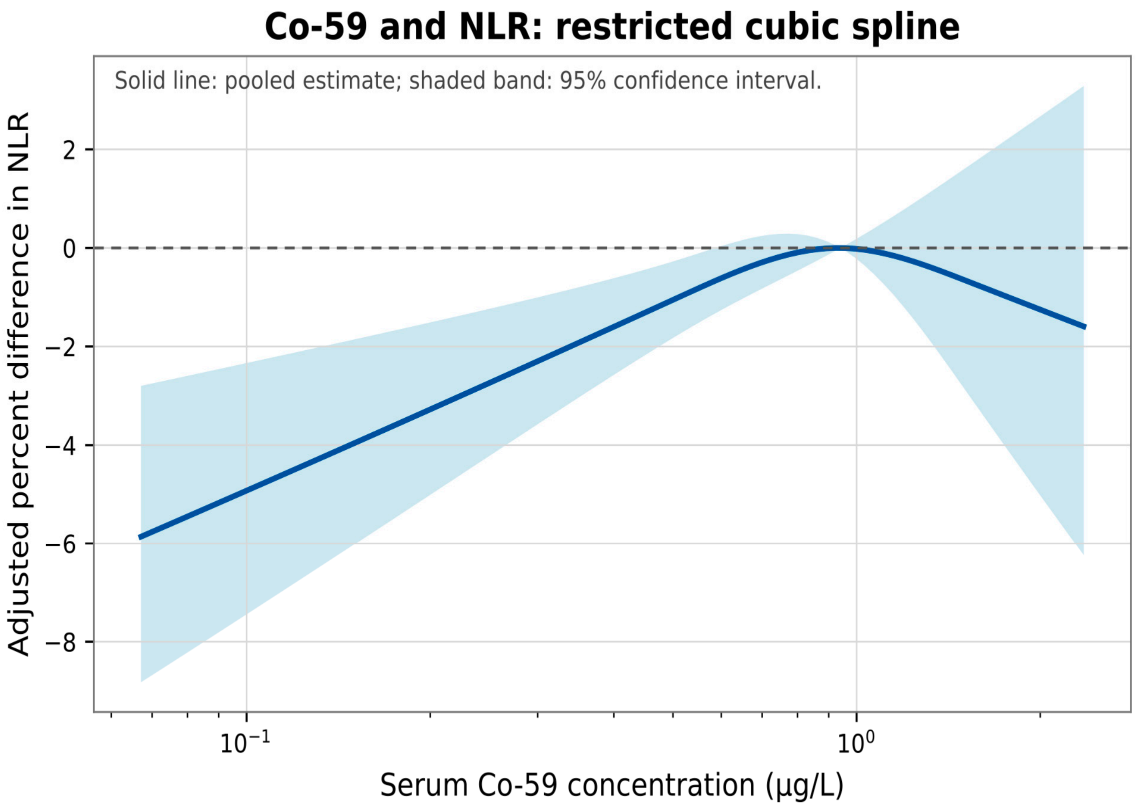

No primary linear element–NLR association met the original outcome-specific FDR threshold or the additional BH threshold across the 38 co-primary comparisons. The secondary global Co spline test was FDR-significant, whereas its specifically nonlinear component was not significant. These are different tests and do not establish the absence of association.

Supplementary Figure S8. Participant flow diagram.

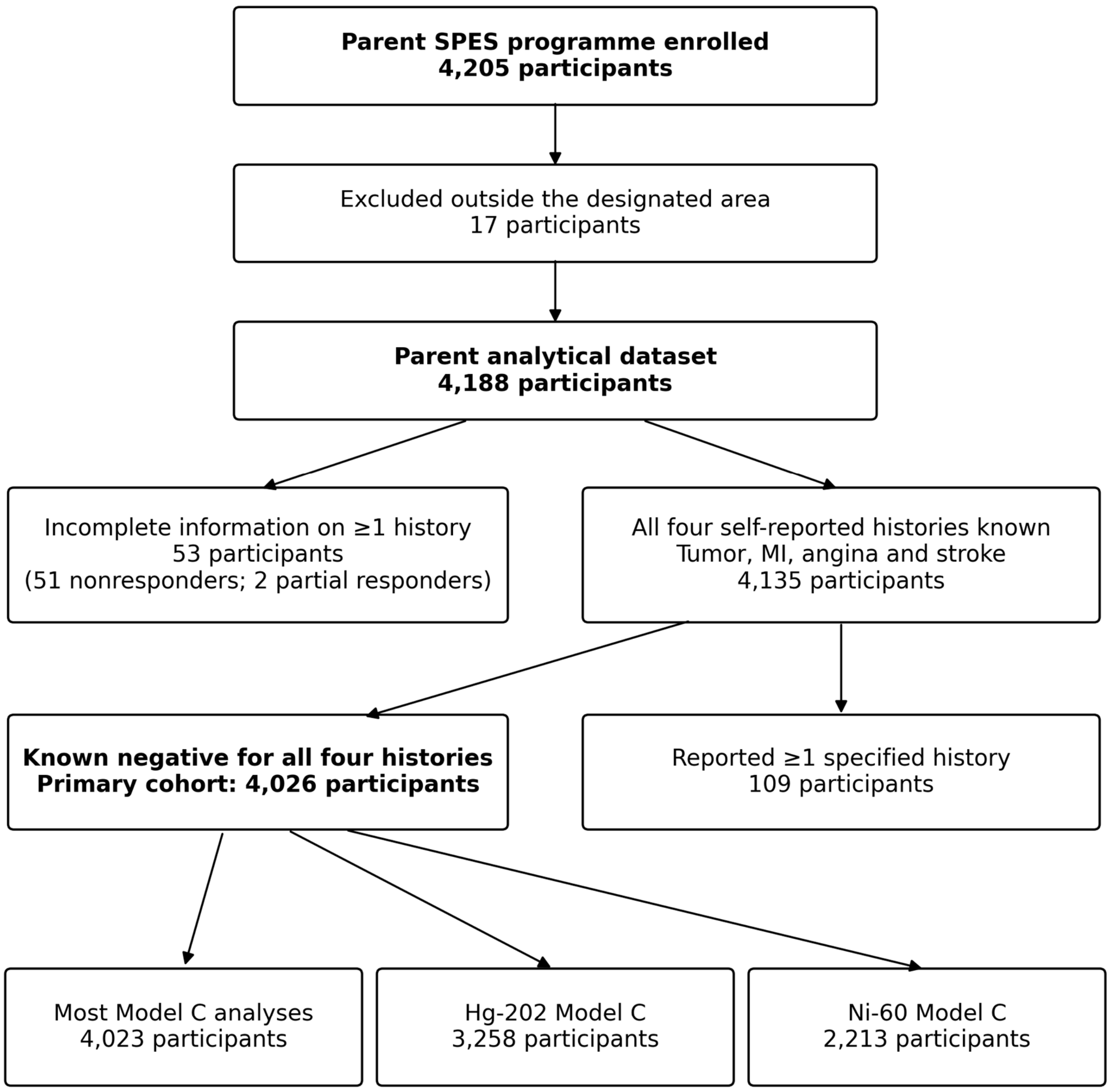

Three participants lacked BMI. Hg and Ni model denominators also reflect assay availability.
MI, myocardial infarction. Histories were self-reported, not clinically validated disease-free status.

Participant flow began with 4,205 enrolled residents. Exclusion of 17 participants recruited outside the designated area yielded 4,188 parent analytical records. Of these, 53 had incomplete information on at least one of the four specified disease histories and 109 reported at least one history, leaving 4,026 participants for the primary analysis. The diagram describes flow among enrolled participants, not the invitation-based participation rate. Disease histories were self-reported. Hg and Ni denominators reflect assay availability.

Supplementary Figure S9. Alternative causal orderings relevant to interpretation of the cross-sectional associations.

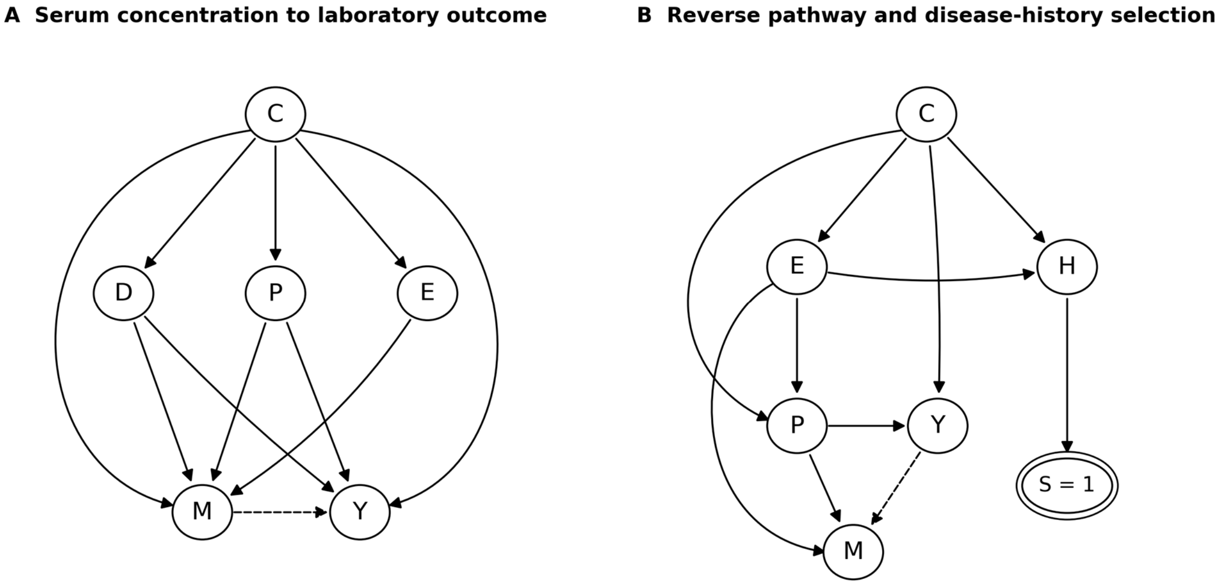

Panel A includes a hypothesized causal pathway from serum element concentration (M) to the laboratory outcome (Y), alongside shared background, nutritional and physiological causes. Panel B includes the alternative Y → M pathway, physiological intermediates of earlier exposure and selection under the disease-history restriction. Solid and dashed arrows both denote causal assumptions, not demonstrated effects; dashed arrows highlight the alternative M–Y directions. The double outline indicates conditioning on inclusion (S = 1). In Panel B, prior disease history (H) is a common consequence of earlier exposure (E) and background causes (C); conditioning on its descendant S can induce selection-related associations. Serum concentrations and laboratory outcomes were measured contemporaneously, so neither temporal ordering is established by these data. C, D and P represent conceptual groups, not a fully measured or diagram-derived adjustment set. Available measures included age, sex, geography, BMI, behavioural and socioeconomic covariates and dietary proxies; creatinine was included only in the renal sensitivity analysis. External source intensity, detailed supplement intake, binding-protein regulation and comprehensive inflammatory physiology were not fully measured. The fitted estimand remains the covariate-adjusted cross-sectional association; neither panel establishes identification of a total or direct causal effect.

Panel A: C, age, sex, geography, behaviour and socioeconomic factors; D, diet and supplements; P, BMI, renal and inflammatory physiology; E, external source intensity; M, serum element concentration; Y, laboratory outcome. Panel B: C, shared background causes; E, earlier external exposure; P, physiological intermediates; M, current serum concentration; Y, current laboratory outcome; H, prior disease history; S, inclusion under the disease-history restriction.

Numbered references in these Supplementary Materials refer to the bibliography of the main article.

Combined STROBE and STROBE-ME Reporting Checklist

Associations of Serum Trace-Element Concentrations with Selected Blood Biomarkers: A Secondary Cross-Sectional Analysis of the SPES Cohort

Cross-sectional human biomonitoring study. Locations use stable section, supplementary-method/result, table and figure identifiers rather than page or line numbers, which may change during production.

STROBE checklist for cross-sectional studies

Section	Item	Recommendation	Reported in
Title and abstract	1(a)	Identify the study design using a commonly used term in the title or abstract.	Main title and Abstract.
Title and abstract	1(b)	Provide an informative and balanced abstract of what was done and found.	Main Abstract.
Background/rationale	2	Explain the scientific background and rationale.	Main Section 1.
Objectives	3	State specific objectives and any hypotheses specified before association modelling.	Main Section 1, objectives. Supplement SM1, specified outcome hierarchy.
Study design	4	Present key elements of the study design early in the paper.	Main Section 2.1. Supplement SM1–SM2.
Setting	5	Describe setting, locations, and relevant dates, including recruitment and data collection.	Main Section 2.1. Supplement SM1 and Figure S8. The data required to reconstruct an invitation-based participation rate were not collected.
Participants	6(a)	Give eligibility criteria and the sources and methods of participant selection for the cross-sectional study.	Main Section 2.1. Supplement SM1–SM2, Table S3 and Figure S8. Eligibility criteria and the observed sample are distinguished.
Participants	6(b)	For matched studies, give matching criteria and numbers in each group.	Not applicable; the study was not matched.
Variables	7	Define outcomes, exposures, predictors, confounders, and effect modifiers; give diagnostic criteria where applicable.	Main Sections 2.2–2.4. Supplement SM3–SM7 and Table S19.
Data sources / measurement	8	For each variable of interest, give data sources and measurement methods; describe comparability across groups.	Main Sections 2.1–2.3. Supplement SM3–SM5, Tables S5 and S19.
Bias	9	Describe efforts to address potential sources of bias.	Main Sections 2.1, 2.2, 2.5 and 4. Supplement SM3, SM5 and Figure S9.
Study size	10	Explain how the study size was determined.	Main Section 2.1, fixed sample size. Supplement Table S6, approximate nominal precision note.
Quantitative variables	11	Explain how quantitative variables were handled and any groupings used.	Main Sections 2.3–2.5. Supplement SM6–SM8 and Table S19.
Statistical methods	12(a)	Describe statistical methods, including control of confounding.	Main Sections 2.4–2.5. Supplement SM7–SM9 and Figure S9. The diagrams illustrate assumptions; causal effects are not identified.
Statistical methods	12(b)	Describe methods used for subgroups and interactions.	No interaction analyses were performed. Main Section 2.5, restrictions and nonlinear analyses. Supplement SM8–SM9 and SR4–SR5.
Statistical methods	12(c)	Explain how missing data were addressed.	Main Sections 2.2, 2.5 and 3.1. Supplement SM3–SM5 and Tables S3–S5.
Statistical methods	12(d)	For cross-sectional studies, describe methods accounting for the sampling strategy.	Main Sections 2.4–2.5 and 4. Supplement SM1 and SM7. Clustering and limits of representativeness are reported.
Statistical methods	12(e)	Describe sensitivity analyses.	Main Sections 2.5 and 3.5. Supplement SM9, Tables S1–S2 and S13–S23. Specified sensitivities and post-primary additions use nominal inference.
Participants	13(a)	Report numbers of individuals at each stage.	Main Sections 2.1 and 3.1. Supplement SM1–SM2, SR1, Table S3 and Figure S8.

Section	Item	Recommendation	Reported in
Participants	13(b)	Give reasons for nonparticipation or exclusion at each stage.	Main Sections 2.1 and 4. Supplement SM1–SM2, Table S3 and Figure S8. The data required to calculate an invitation-based participation rate and systematically compare participants with nonparticipants were not collected.
Participants	13(c)	Consider use of a flow diagram.	Supplement Figure S8, participant flow.
Descriptive data	14(a)	Report participant characteristics, exposures, and potential confounders.	Main Section 3.1 and Table 1. Supplement SR1 and Tables S4 and S18. The structured examination findings use assessment-specific denominators.
Descriptive data	14(b)	Report numbers with missing data for variables of interest.	Main Section 3.1 and Table 1. Supplement SM3–SM5 and Tables S3–S5. Original-record availability and imputation status are distinguished.
Descriptive data	14(c)	For cohort studies, summarize follow-up time.	Not applicable to this cross-sectional analysis.
Outcome data	15	Report outcome numbers or summary measures for the cross-sectional study.	Main Table 1. Supplement Table S4.
Main results	16(a)	Report unadjusted estimates and, if applicable, confounder-adjusted estimates with precision; identify adjusted confounders and the rationale for inclusion.	Main Section 2.4, covariates. Main Sections 3.2–3.5 and Tables 2–3. Supplement SM7 and complete results, Tables S6–S24. Models A–C were progressively adjusted; fully unadjusted estimates were not presented.
Main results	16(b)	Report category boundaries when continuous variables were categorized.	Primary exposures were continuous. Main Section 3.5, quantification-status contrasts. Supplement SM3, SM9 and Tables S5 and S19.
Main results	16(c)	When relevant, translate relative estimates into absolute terms.	Main Sections 3.2 and 3.4. Supplement SM6 and Tables S13, S19 and S23. Native-unit regression estimates and standardized geometric-mean contrasts are distinguished.
Other analyses	17	Report other analyses, including sensitivity analyses.	Main Sections 2.5 and 3.5. Supplement SM8–SM9, SR4–SR7 and Tables S12–S24.
Key results	18	Summarize key results with reference to study objectives.	Main Sections 4 and 5.
Limitations	19	Discuss limitations, including potential bias or imprecision and likely direction/magnitude.	Main Section 4. Supplement SM3–SM5, SM7 and SR2–SR7.
Interpretation	20	Provide a cautious overall interpretation considering objectives, limitations, multiplicity, and other evidence.	Main Sections 4–5. Supplement SM8–SM9 and SR4–SR7.
Generalizability	21	Discuss external validity.	Main Sections 2.1 and 4. Supplement SM1–SM2 and Table S3. Restrictions and the absence of systematically collected nonparticipant information limit assessment of representativeness.
Funding	22	State funding sources and the role of funders.	Main Funding.

STROBE-ME extension for molecular epidemiology

Only STROBE-ME items that add molecular-epidemiology-specific recommendations to the parent STROBE items are listed; intervening items are covered by the STROBE table above. Reporting guidelines used: STROBE cross-sectional checklist and STROBE-ME extension. This checklist is a reporting aid and not an instrument for grading methodological quality.

Item	STROBE-ME recommendation	Reported in
ME-1	State use of specific biomarkers in the title and/or abstract when they contribute substantially.	Main title and Abstract.
ME-2	Explain why the specific biomarkers were selected and what they represent.	Main Sections 1 and 2.3. Supplement SM3–SM4.
ME-3	State the a priori biomarker hypothesis when biomarkers are used as proxy measures.	Main Section 1, objectives. Supplement SM1 and SM8, specified outcome hierarchy.

Item	STROBE-ME recommendation	Reported in
ME-4	Describe any special molecular-epidemiology design and its implementation.	Conventional secondary cross-sectional analysis. Main Section 2.1. Supplement SM1–SM2.
ME-4.1	Report the setting and conditions of biological sample collection, amount, collection procedures, participant conditions, and timing relative to relevant endpoints.	Main Sections 2.1–2.2. Supplement SM2–SM4. Published fasting instruction/staff check, same-encounter timing and 500 µL aliquot are reported; individual fasting duration and sampling-time records were unavailable.
ME-4.2	Describe biological sample processing, including centrifugation, timing, and additives.	Main Section 2.2. Supplement SM3. Exact processing intervals were not documented.
ME-4.3	Describe sample storage until biomarker analysis, including storage, thawing, and manipulation.	Main Section 2.2. Supplement SM3.
ME-4.4	Report relevant biomarker half-life and chemical/physical characteristics.	Main Sections 2.2 and 4. Supplement SM3 and SM6. Matrix, time-window and speciation limitations are reported; element-specific half-lives were not used to infer lifetime body burden.
ME-6	Report habits, clinical conditions, physiological factors, and working/living conditions that may affect biomarker concentrations.	Main Sections 2.1, 2.3–2.4, 3.1 and Table 1. Supplement SM1–SM2, SM4, SM7, SR1 and Tables S4 and S18.
ME-8	Report laboratory methods, assay type, detection limits, outliers, calibration, standards, and relevant assay timing.	Main Section 2.2. Supplement SM3 and Table S5.
ME-12	Describe how biomarkers were introduced into statistical models.	Main Sections 2.3–2.5. Supplement SM6–SM8.
ME-12.1	Report evidence on biomarker measurement validity/reliability and internal or external validation.	Main Sections 2.2 and 4. Supplement SM3–SM5 and Table S5. Published standards and blank-based LOQs are reported; numerical precision, analyte-specific recovery and raw replicate summaries were unavailable.
ME-13	Give reasons for loss of biological samples at each stage.	Main Sections 2.1–2.2 and 3.1. Supplement SM3, Tables S5 and S16, and Figure S8. Nonperformance, below-LOQ observations and QC-high flags are distinct; operational reasons for Hg/Ni nonperformance were unavailable.
ME-14.1	Report the distribution of biomarker measurements, including mean, median, range and variance.	Main Section 3.1 and Table 1. Supplement Tables S4–S5 and S19. Descriptive medians/IQRs and approximate Q1–Q3 concentration summaries are reported; empirical means, full ranges and variances are not.
ME-19	Describe major limitations of laboratory procedures.	Main Section 4. Supplement SM3–SM5 and SR2.
ME-20	Interpret results in terms of a priori biological plausibility.	Main Section 4. Supplement SR7.
ME-22.1	Describe informed consent and ethics approval; state whether samples/data were anonymous, anonymized, or identifiable.	Main Section 2.6 and ethics/consent statements. The analytical extract was de-identified.
